# Sleeve Gastrectomy Improves Metabolic Health, Cognition, and Alzheimer's Disease Pathology in 3xTG Mice

**DOI:** 10.1111/acel.70767

**Published:** 2026-10-13

**Authors:** Reji Babygirija, Julia A. Illiano, Michelle M. Sonsalla, Grace Zhu, Christopher Retter Jabbarpour, Twinkle Mathew, Tristan Molkentin, Meredith Peterson, Carolyn Winder, Advaith Ravi Krishnan Nair, Fan Xiao, Justin M. Wolter, Dudley W. Lamming, David A. Harris

**Affiliations:** ^1^ Wisconsin Surgical Laboratory in Metabolism, Department of Surgery University of Wisconsin‐Madison Madison Wisconsin USA; ^2^ William S. Middleton Memorial Veterans Hospital Madison Wisconsin USA; ^3^ Department of Medicine University of Wisconsin‐Madison Madison Wisconsin USA; ^4^ Laboratory of Genetics University of Wisconsin‐Madison Madison Wisconsin USA; ^5^ University of Wisconsin‐Madison Comprehensive Diabetes Center Madison Wisconsin USA; ^6^ Wisconsin Nathan Shock Center of Excellence in the Basic Biology of Aging Madison Wisconsin USA

**Keywords:** Alzheimer's disease, autophagy, bariatric surgery, insulin signaling, neuropathology, sleeve gastrectomy

## Abstract

Obesity and diabetes are well‐established risk factors for Alzheimer's disease (AD), implicating metabolic dysfunction in AD pathogenesis. Sleeve gastrectomy (SG) is among the most effective metabolic interventions available, yet its impact on AD progression remains poorly understood. We hypothesized that SG performed early in life would improve metabolic health, attenuate AD pathology, and preserve cognition in a transgenic AD mouse model. Five‐week‐old female 3xTg‐AD mice were preconditioned on a Western diet (WD) to induce obesity and glucose intolerance, then randomized to SG or sham surgery and maintained on either Control or continued WD for 12 months. Metabolic phenotyping, body composition, and cognitive assessments (Novel Object Recognition, Barnes Maze, Fear Conditioning) were performed longitudinally, with histological and molecular brain analysis at endpoint. Under Control‐fed conditions, SG reduced fat mass and improved peripheral insulin sensitivity independent of food intake. These changes were accompanied by reduced cortical Aβ plaque burden and attenuated astrocytic (GFAP) and microglial (IBA1) activation, specifically in the cortex. Mechanistically, SG altered the insulin signaling‐autophagy axis in a region‐specific manner, with increased pAKT and suppressed ATG5/ATG7/ATG16L1 in the cortex. Under persistent WD, SG improved glucose homeostasis, reduced fat mass, lowered frailty, and rescued short‐ and long‐term spatial memory deficits in the Barnes maze, accompanied by reduced cortical Aβ burden. Collectively, these findings provide the first preclinical evidence that SG attenuates AD progression through a brain insulin signaling‐autophagy axis, supporting metabolic surgery as a promising disease‐modifying intervention for AD prevention in individuals with obesity.

## Graphical abstract

Through a single metabolic intervention, Sleeve Gastrectomy improves metabolic health and attenuates Alzheimer's pathology in 3xTg‐AD mice across dietary contexts. It reduces cortical amyloid and neuroinflammation via region‐specific brain insulin–autophagy signaling and rescues memory under Western‐diet stress, independent of caloric intake.

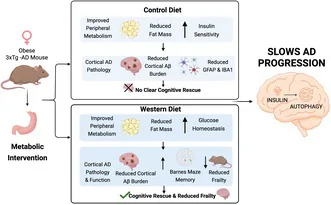

## 1 Introduction

Alzheimer's Disease (AD) represents one of the most urgent public health crises of the 21st century. As the global population ages, the prevalence of AD is growing at an alarming rate. Approximately 6.9 million Americans aged 65 and older are currently living with the disease, a number projected to nearly double to 13.8 million by 2060 in the absence of any effective preventive or curative treatments (Anon 2024). While recent therapeutic advances mildly impact AD progression, these treatments are associated with significant side effects, high cost, and limited efficacy, highlighting the urgent need for alternative, accessible, and affordable therapeutic strategies for AD.

Metabolic dysfunction is increasingly recognized as a critical contributor to AD pathology. Obesity and diabetes are well‐established risk factors for AD, with evidence linking insulin resistance, systemic inflammation, and metabolic dysregulation to neurodegeneration (Khan and Hegde 2020; Pugazhenthi et al. 2017). Beyond cardiovascular complications, accumulating evidence demonstrates that excess adiposity is independently associated with increased risk of dementia and cognitive impairment across the lifespan (Fitzpatrick et al. 2009; Fergenbaum et al. 2009). Targeted dietary interventions including caloric restriction, protein restriction and intermittent fasting have been shown to influence AD progression in mice (Halagappa et al. 2007; Rangan et al. 2022; Babygirija et al. 2024, 2025; Babygirija, Green, et al. 2026; Kapogiannis et al. 2024). However, the long‐term sustainability of such interventions remains a significant challenge as adherence to these dietary regimens are consistently low in humans. Additionally, novel incretin‐based therapies targeting the glucagon‐like peptide 1 (GLP‐1) and gastric inhibitory polypeptide (GIP) receptors have been extensively studied in this context with mixed results in mice (Holscher 2021; Germano et al. 2024; Maskery et al. 2020) and have not been shown to modify disease progression in humans (Cummings et al. 2025; Scheltens et al. 2026).

Among metabolic interventions, bariatric surgery (BS) remains the most effective and durable treatment for substantial, rapid weight loss and resolution of obesity‐associated comorbidities (Gloy et al. 2013; Mingrone et al. 2021). Unlike other metabolic interventions mentioned above, BS is a one‐time intervention with lifelong impact. Sleeve gastrectomy (SG) is now the most performed bariatric procedure worldwide (Gagner et al. 2016). While SG works in part through the induction of incretin hormones to impart metabolic benefits (Chaudhari et al. 2021), incretin signaling is not required to induce weight loss and improvement in diabetes (Wilson‐Pérez et al. 2013), suggesting BS engages a broad array of gut–brain pathways. Furthermore, the benefits of BS extend well beyond metabolic improvements. Neuroimaging studies have demonstrated structural and functional brain changes following BS (Almby et al. 2021; Tuulari et al. 2016; Zhang et al. 2016) and epidemiological data indicate that BS is associated with significant improvements in cognitive function as early as 12 weeks post‐operatively, with sustained benefits observed through 24 months (Spitznagel et al. 2013; Gunstad et al. 2011). Remarkably, a recent retrospective cohort study reported that BS was associated with reduced incidence of mild cognitive impairment and AD‐related dementias (Chen et al. 2025). Despite these promising findings on cognition and metabolic dysfunction, the impact of BS on AD‐specific pathology and the mechanisms driving this neuroprotection remain unknown.

Diet has been identified as one of the key modulators of AD pathology. Long‐term consumption of a western diet (WD) has been previously shown to exacerbate multiple aspects of AD pathology, including neuroinflammation, β‐amyloid pathology, and tau pathology in transgenic mouse models of AD (Sonsalla et al. 2025; Graham et al. 2016; Wiȩckowska‐Gacek et al. 2021). Importantly, prior work from our laboratory in C57BL/6J mice demonstrated that a low‐protein diet following SG sustained the metabolic benefits of surgery and identified clusters of differentially expressed genes and metabolites associated with the weight‐loss phenotype, with effects primarily driven by hepatic tissue (Illiano et al. 2025). However, the downstream effects of SG on AD‐related phenotype and cognitive function in the context of diet have not been previously studied.

To address this gap, we used the 3xTg mouse model, which expresses familial human isoforms of APP (APPSwe), Tau (tauP301L), and Presenilin (PS1M146V), and exhibits key features of human AD, including Aβ plaques, neurofibrillary tangles, and progressive cognitive deficits (Webster et al. 2014; Oddo et al. 2003). Female 3xTg‐AD mice were preconditioned on a WD for 2 months prior to undergoing SG or sham surgery. They were then maintained on one of two lifelong diets: standard Control (Envigo TD.180161; 21% kcal from protein, 20% from fat, 58.8% from carbohydrates) or WD (Envigo TD.88137; 42% kcal from fat, 15% from protein, 43% from carbohydrates; sucrose as primary carbohydrate source). This experimental design allowed us to evaluate the effects of SG both under baseline metabolic conditions and in the context of diet‐induced metabolic stress.

In the present study, we tested the central hypothesis that SG would attenuate AD progression in 3xTg‐AD mice by improving overall metabolic health, reducing neuroinflammation, and decreasing Aβ pathology across dietary contexts. Our results demonstrate that SG reduced adiposity, improved insulin sensitivity, and was associated with reduced cortical amyloid plaque burden and glial activation in Control‐fed mice. In mice maintained on a WD, SG had additional benefits including improved spatial learning and memory and reduced plaque pathology. Taken together, these findings provide the first preclinical evidence that SG can slow AD progression in a transgenic AD mouse model, highlighting the potential of metabolic surgery as a disease‐modifying intervention for AD and warranting further clinical investigation.

## 2 Results

### 2.1 SG Reduces Adiposity and Improves Insulin Sensitivity Without Altering Energy Balance in Control‐Fed 3xTg‐AD Mice

Five‐week‐old female 3xTg‐AD and wild‐type (WT) mice were preconditioned on WD and weight‐matched prior to randomization into Sham or SG surgery groups. WD preconditioning was used to induce obesity and glucose intolerance prior to surgery, recapitulating the metabolic comorbidities commonly present in patients seeking BS. Following surgery, all mice were transitioned to a standard Control diet mimicking the post‐operative dietary recommendations in clinical practice. Body weight, food intake, and body composition were tracked longitudinally for 12 months post‐surgery, with body composition assessed at baseline and endpoint (Figure 1A).

**FIGURE 1:**
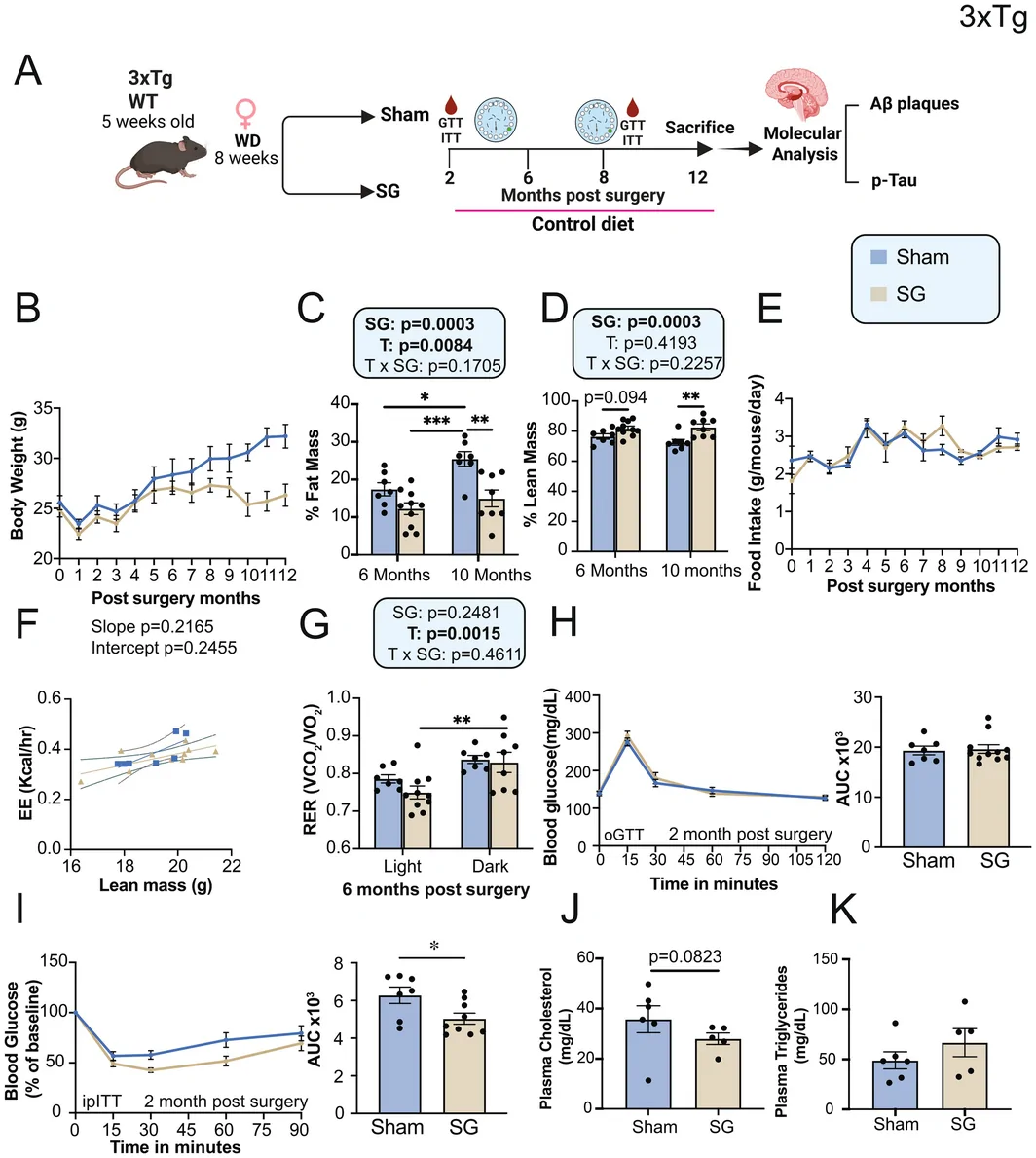
Sleeve gastrectomy reduces adiposity and improves insulin sensitivity independent of food intake in Control‐fed 3xTg‐AD mice. (A) Experimental design. Female 3xTg‐AD and wild‐type (WT) mice were preconditioned on Western diet (WD) for 8 weeks prior to weight‐matched randomization to receive either Sham or Sleeve gastrectomy (SG). They were then transitioned to control diet post‐surgery. GTT and ITT were performed at 2 months post‐surgery. Schematic created with www.biorender.com. (B) Longitudinal bodyweight of female 3xTg‐AD mice over 12 months post‐surgery. (C) Percent fat mass (D) percent lean mass as assessed by echoMRI body composition analysis at 6 and 10 months post‐surgery. (E) Average daily food intake monitored over 12 months post‐surgery. (F) Energy expenditure (EE) normalized to lean mass at 6 months post‐surgery. (G) Respiratory exchange ratio (RER) during light and dark phases at 6 months post‐surgery. (H) Oral glucose tolerance test (oGTT) performed at 2 months post‐surgery with corresponding area under the curve (AUC). (I) Intraperitoneal insulin tolerance test (ipITT) performed at 2 months post‐surgery with corresponding AUC. (J) Plasma total cholesterol and (K) plasma triglycerides measured at endpoint in 3xTg‐AD Sham and SG mice. (C, D, F, G) statistics show the overall effects of surgery (SG), time (T), and their interaction (T × SG) from a two‐way ANOVA; with *p < 0.05, **p < 0.01, ***p < 0.001, from Sidak's multiple comparison test. (H, I) group differences in AUC were assessed by Welch's *t*‐test; *p < 0.05, (B–I) *n* = 8–10/group and (J) *n* = 5/group. Data represented as mean ± SEM. *n* per group shown as individual data points.

Compared to Sham mice, SG 3xTg‐AD mice trended toward lower body weight, which became more pronounced over the course of the 12‐month study period (Figure 1B). This was driven primarily by a significant reduction in fat mass at 6 and 10 months post‐surgery (Figure 1C). Lean mass was significantly greater in SG mice at 10 months post‐surgery, with a trend toward greater lean mass at 6 months (*p* = 0.094; Figure 1D). To determine whether reduced food intake accounted for the lower body weight, we examined 12 months of cumulative feeding data. Consistent with our prior work (Harris et al. 2020; Chaudhari et al. 2021; Illiano et al. 2025), caloric intake did not differ between SG and Sham groups (Figure 1E), indicating that reduced caloric intake did not drive the observed differences in body weight and adiposity. WT mice showed analogous reductions in body weight and adiposity without differences in food intake (Figure S1A–E), confirming that the metabolic benefits of SG on body composition are independent of both the AD transgenic background and caloric restriction.

Since SG mice gained less weight than sham mice despite equivalent food intake, we examined energy balance using metabolic chambers. We assessed substrate utilization via the respiratory exchange ratio (RER), calculated as the ratio of CO_2_ produced to O_2_ consumed. RER approaches 1.0 when carbohydrates are the primary fuel source and 0.7 when lipids predominate. Surprisingly, surgery had no significant effect on energy expenditure (EE) or RER at 6 or 10 months post‐surgery (Figure 1F–G; Figure S2A,B). However, a significant effect of time on RER was observed during the dark phase in SG mice at 6 months (Figure 1G), suggesting a transient shift toward greater glucose utilization during the active phase. WT SG mice similarly showed no surgery‐driven differences in RER between light and dark phases relative to WT sham controls (Figure S1E–F), indicating this effect is independent of the AD transgenic background. Together, these data demonstrate that reduced adiposity in SG mice is not attributable to differences in total EE or substrate utilization.

Given that 3xTg‐AD mice exhibit progressive impairment in glucose tolerance (Babygirija et al. 2024; Vandal et al. 2015), we examined the impact of SG on glucose handling via an oral glucose tolerance test (oGTT) at 2 months post‐surgery. This revealed no significant differences in glucose excursion or area under the curve (AUC) between SG and Sham groups in either 3xTg or WT mice (Figure 1H; Figure S1G). This finding contrasts with our prior work in non‐transgenic C57BL/6J mice, where SG robustly improved glucose tolerance across all dietary conditions (Chaudhari et al. 2021; Harris et al. 2020; Illiano et al. 2025). Nevertheless, an intraperitoneal insulin tolerance test (ipITT) at the same timepoint revealed significantly improved insulin sensitivity in 3xTg‐SG mice relative to shams, an effect not observed in WT mice (Figure 1I; Figure S1H). These findings suggest that SG improves peripheral insulin sensitivity in 3xTg‐AD mice independent of glucose tolerance, likely reflecting compensatory adaptations specific to age and the transgenic background.

To assess whether SG impacted baseline incretin signaling, total fasting GLP‐1 levels were measured in plasma samples from all groups. There was an overall significant effect of surgery on fasting plasma GLP‐1 levels (SG: *p* = 0.0487), with SG WT mice showing significantly elevated GLP‐1 compared to sham WT controls. However, we did not observe a significant increase in fasting plasma GLP‐1 concentration in 3xTg‐AD mice (Figure S3A), which may differ from the post‐prandial state.

To further characterize the post‐surgical metabolic profile, plasma total cholesterol and triglyceride levels were assessed in both 3xTg‐AD and WT mice. In keeping with our prior work (Babygirija, Illiano, et al. 2026), there was a trend toward lower plasma cholesterol in SG 3xTg‐AD mice and WT mice (Figure 1J; Figure S1I). There were no differences in plasma triglycerides observed across genotypes (Figure 1K; Figure S1J).

Collectively, these data demonstrate that SG reduces adiposity and improves insulin sensitivity in Control‐fed female 3xTg‐AD mice without altering food intake or energy balance, with the body weight effect mediated primarily through changes in body composition.

### 2.2 SG Attenuates AD Neuropathology by Reducing Cortical Plaque Deposition and Glial Activation

Having established that SG improves metabolic health in Control‐fed 3xTg‐AD mice, we next examined whether these benefits extended to AD neuropathology. We evaluated key pathological hallmarks including Aβ plaque deposition, tau phosphorylation, and gliosis. Brain sections from 15‐month‐old female 3xTg‐AD mice were immunostained for Aβ plaques using 3,3′‐diaminobenzidine (DAB) and imaged across multiple magnifications in the cortex and hippocampus (Figure 2A). Quantification revealed a significant reduction in cortical plaque percent area (SG: *p* = 0.0268) and a trend toward reduced plaque number (*p* = 0.0547) in SG mice relative to shams (Figure 2B). Although there appeared to be reduced plaque burden within the hippocampus, this did not reach statistical significance, indicating that the anti‐amyloidogenic effects of SG are cortex‐predominant at 15 months (Figure 2B, upper and lower, respectively).

**FIGURE 2:**
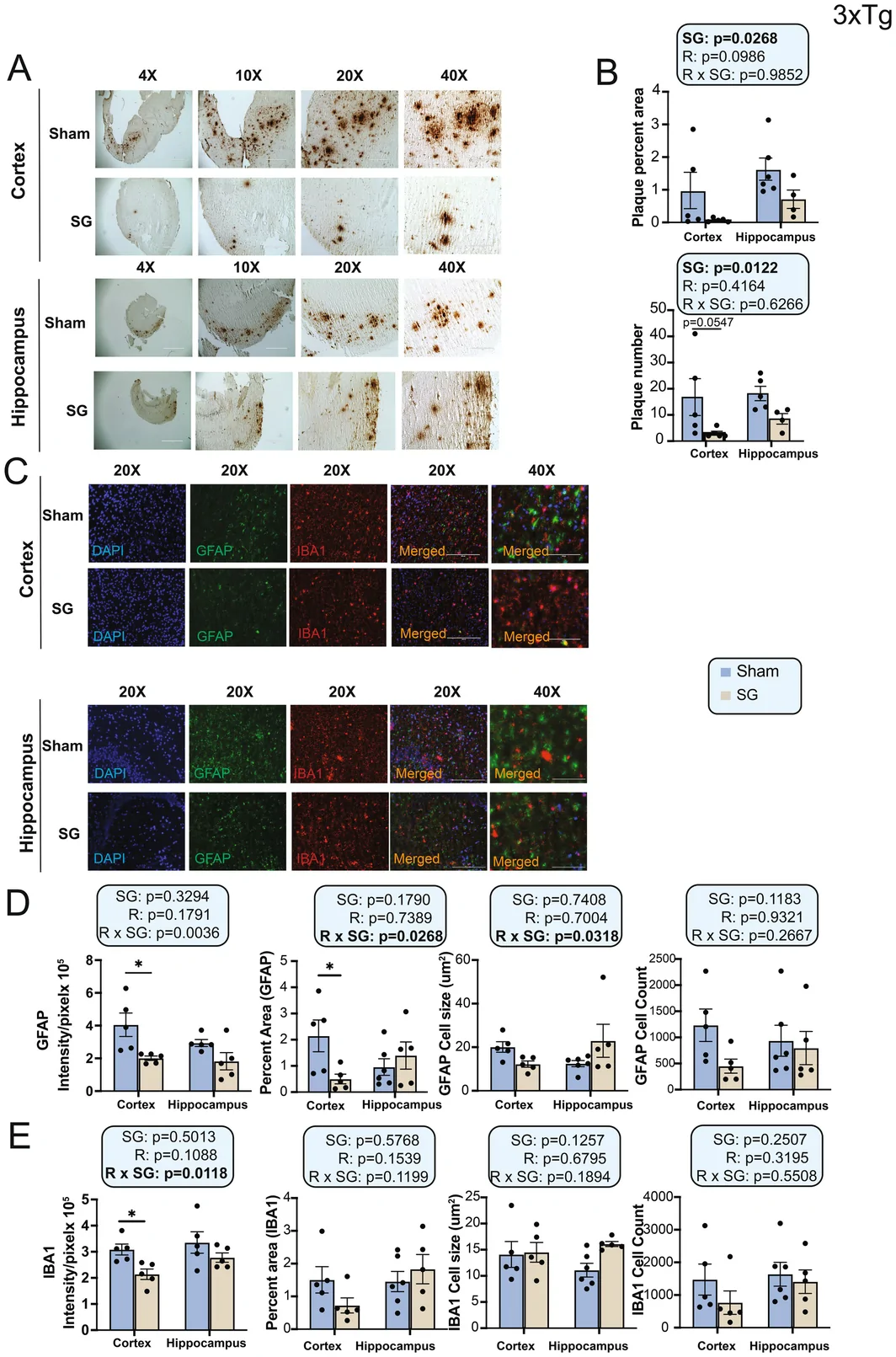
Sleeve gastrectomy reduces cortical amyloid plaque burden and glial activation in Control‐fed 3xTg‐AD mice. (A) Representative DAB immunostaining images of Abeta plaques in cortical (top) and hippocampal (bottom) sections from 15‐month‐old female 3xTg‐AD mice. 4×, 10×, 20×, and 40× magnification shown; scale bar in the 4× image is 1000 μM, 10× image is 400 μM, 20× is 200 μM, and 40× is 100 μM. (B) Quantification of plaque percent area (upper) and plaque number (lower) in cortex and hippocampus. Two non‐adjacent tissue sections per animal were analyzed with the entire cortical and hippocampal regions quantified. Values averaged across sections per animal. (C) Representative immunofluorescence images of 5 μm paraffin‐embedded brain sections co‐stained for astrocytes (GFAP‐green) and microglia (IBA1‐red) at 20× and 40× magnification. Scale bar = 200 μm. (D) Quantification of GFAP immunolabeling showing mean intensity (×10^5^), percent area, average cell size (μm^2^), and cell count in cortex and hippocampus. (E) Quantification of IBA1 immunolabeling showing mean intensity (×10^5^), percent area, average cell size (μm^2^), and cell count in cortex and hippocampus. (B, D, E) statistics show the overall effects of surgery (SG), brain region (R), and their interaction (*R* × SG) from a two‐way ANOVA; Mann Whitney *U* tests were additionally performed within each brain region to directly compare SG vs. Sham groups. Results are summarized in Table S2. *p < 0.05, **p < 0.01 from Sidak's multiple comparison test. (A, B) *n* = 5 Sham, *n* = 4 SG; (C–E) *n* = 5/group. Data represented as mean ± SEM.

We next assessed tau pathology by immunoblotting for a panel of phosphorylated tau epitopes including pThr231, AT8 (pSer202/Thr205), pSer409, and pSer199, normalized to total tau (Figure S4A–E). The effects of SG on tau phosphorylation were epitope‐specific and did not follow a uniform pattern. AT8 immunoreactivity was significantly elevated in the cortex of SG mice relative to shams (Figure S4C), while pThr231 in the cortex did not differ significantly between groups. (*p* = 0.4586; Figure S4B). pSer409 and pSer199 did not differ between groups and regions (Figure S4D,E). There was a strong trend toward reduced pThr231 within the cortex and no change in pThr231 in the hippocampus of 3xTg SG mice as measured by immunofluorescence staining for pThr231 (*p* = 0.0550; Figure S4F). Together these data suggest that SG differentially modulates tau phosphorylation in an epitope‐ and region‐specific manner, with effects most pronounced in the cortex.

Finally, we assessed reactive astrogliosis and microglial activation, which are both hallmark features of AD neuroinflammation (Bronzuoli et al. 2019; Adamu et al. 2024; Kiraly et al. 2023). Brain sections were co‐immunostained for glial fibrillary acidic protein (GFAP), a marker of reactive astrocytes, and ionized calcium‐binding adaptor molecule 1 (IBA1), a marker of activated microglia (Figure 2C). Quantification of mean intensity, percent area, cell count, and average cell size were performed for both markers in the cortex and hippocampus (Figure 2D,E). In the cortex, there was a significant reduction in GFAP staining intensity and percent positive area in SG mice relative to shams, consistent with the pattern of reduced plaque burden within the cortex. There was a similar reduction in IBA1 staining intensity and a trend toward reduced total percent area of IBA1 staining in the SG cortex. However, there were no differences in GFAP or IBA1 detected in the hippocampus across any measure, demonstrating a clear region‐specific attenuation of neuroinflammation.

Collectively, these findings demonstrate that SG significantly attenuates multiple hallmarks of AD neuropathology in Control‐fed 3xTg‐AD mice—including Aβ plaque deposition and glial activation—with effects predominating in the cortex.

### 2.3 SG Differentially Regulates Autophagy Machinery and Insulin Signaling in a Region‐Specific Manner

Impaired autophagy results in ineffective clearance of plaques and tangles, accelerating disease progression in both patients and animal models (Pickford et al. 2008; Nixon et al. 2005). Given the significant reductions in cortical plaque burden and gliosis observed in SG mice, we investigated whether SG altered autophagy pathway activity in the brain. Cortical and hippocampal lysates from 15‐month‐old female 3xTg‐AD mice were immunoblotted for a panel of autophagy markers: the initiation proteins ATG5, ATG7, and ATG16L1; the autophagosome‐associated lipidation marker LC3A/B; and the cargo receptor p62/SQSTM1, whose accumulation inversely reflects autophagic flux. Phosphorylated AKT (pAKT), an upstream inhibitor of autophagy initiation via mechanistic target of rapamycin complex 1 (mTORC1) activation, was also assessed (Figure 3A).

**FIGURE 3:**
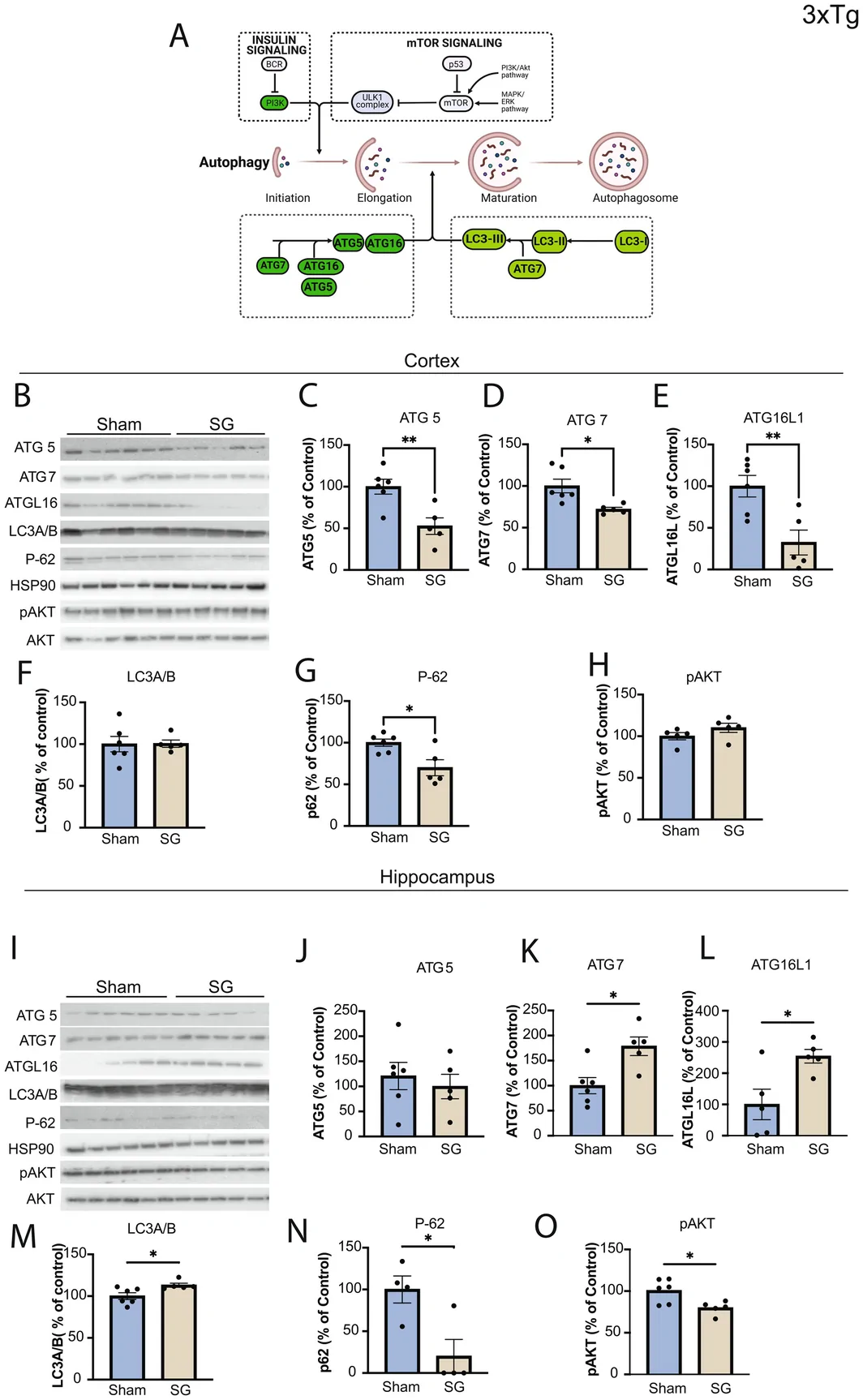
Sleeve gastrectomy differentially regulates autophagy machinery and insulin signaling in a brain‐region‐specific manner in Control‐fed 3xTg‐AD mice. (A) Schematic of the autophagy pathway. (B–H) Autophagy marker expression in cortical lysates of 15‐month‐old female 3xTg‐AD mice following SG or sham surgery. (B) Representative immunoblots of the autophagy related proteins in the cortex. Quantification of autophagy proteins (C) ATG5, (D) ATG7, and (E) ATG16L1, as well as autophagosome formation protein (F) light chain 3A/B (LC3A/B), and the autophagy receptor (G) p62 (sequestosome 1, SQSTM1), and (H) pAKT, all normalized to HSP90 expression. (I) Representatives immunoblot of all the autophagy‐related proteins in the hippocampus. Quantification of ATG5 expression (J), ATG7 (K), ATG16L1 (L), LC3A/B (M), p62 (N), and pAKT (O) relative to expression of HSP90. (C–H, J–O) *p < 0.05, **p < 0.01, Sham and SG groups were compared using Welch's *t*‐test. Data represented as mean ± SEM.

In the cortex, SG mice exhibited significant downregulation of autophagy initiation markers relative to shams (Figure 3B); specifically, ATG5 (Figure 3C), ATG7 (Figure 3D), and ATG16L1 (Figure 3E) were each significantly reduced, indicating suppression of early autophagosome formation. LC3A/B levels were unchanged between groups (Figure 3F), while p62 was significantly reduced (Figure 3G). Rather than reflecting enhanced autophagic flux, the reduction in cortical p62 most likely reflects decreased substrate availability consequent to the significant attenuation of cortical amyloid burden in SG mice. This interpretation is supported by the concurrent downregulation of ATG5, ATG7, and ATG16L1, collectively consistent with a homeostatic reduction in autophagic demand in a region where pathological load has been substantially cleared. A trend toward increased pAKT in SG mice (Figure 3H) further supports suppression of autophagy initiation via mTORC1 activation.

In contrast, the hippocampus displayed a strikingly divergent pattern (Figure 3I). SG mice showed no change in ATG5 (Figure 3J) but significant upregulation of ATG7 (Figure 3K) and ATG16L1 (Figure 3L), collectively indicating enhanced autophagy initiation. This was accompanied by increased LC3A/B (Figure 3M) and markedly reduced p62 (Figure 3N), indicating augmented autophagosome formation, enhanced flux, and effective cargo clearance. Concordantly, pAKT was significantly reduced in the hippocampus of SG mice (Figure 3O), consistent with relief of mTORC1‐mediated autophagy suppression.

Collectively, these data reveal a striking region‐specific divergence in autophagy regulation following SG, likely mediated through the AKT/mTORC1 pathway. The trend toward increased cortical pAKT in SG mice is consistent with the improved peripheral insulin sensitivity observed at this timepoint, suggesting that systemic improvements in insulin signaling may extend to the brain and contribute to region‐specific modulation of mTORC1‐mediated autophagy. The region‐specific divergence may also reflect the differential plaque burden across regions. In the cortex, where SG reduced plaque burden, autophagy is correspondingly downregulated, whereas in the hippocampus, where plaque burden remains elevated, autophagy is upregulated, potentially representing a compensatory response to greater pathological load.

### 2.4 SG Does Not Improve Cognition in Control‐Fed 3xTg‐AD Mice

We next examined whether the neuropathological benefits of SG translated into cognitive improvements by performing a battery of behavioral tests at 5 months (9 months of age) and 8 months (13 months of age) post‐surgery. Tests included spatial learning and memory (Barnes Maze; BM), associative fear memory (contextual and cued fear conditioning; FC), and object recognition memory (Novel Object Recognition; NOR). In the BM, mice were trained over 4 days to locate a hidden escape box using spatial cues, with short‐term memory (STM) and long‐term memory (LTM) assessed on Days 5 and 12, respectively. In FC, mice were placed in a novel chamber and exposed to an auditory tone followed by a 2 s mild foot shock during training. Associative memory was assessed 24 h later in two phases: contextual fear memory was tested by re‐exposing the mice to the original training chamber in the absence of tone, and the cued memory test was assessed in a novel context with tone presentation. Freezing behavior was recorded as the measure of fear memory in both these phases. In the NOR test, which exploits a mouse's natural preference for novelty, exploration of novel and familiar objects was quantified via the discrimination index (DI)—calculated as the difference in time exploring the novel versus familiar object divided by total exploration time—at both STM and LTM recall. A positive DI indicates preferential exploration of the novel object and is interpreted as consolidated memory.

At 5 months post‐surgery (9 months of age), SG and sham mice showed comparable acquisition of the escape hole location across the 4‐day BM training phase, with no significant differences in latency to goal or error hole visits at either STM or LTM (Figure 4A,B). Path length traveled during the training phase similarly did not differ between SG and sham groups across all four training days in either 3xTg‐AD or WT mice (Figure S5A). 5 months following surgery there was a non‐significant trend toward reduced distance traveled during the STM testing in SG mice (*p* = 0.0773; Figure S5A). Thus, confirming that the absence of group differences in BM latency was not attributable to differences in locomotor velocity. In the FC paradigm, no significant surgery‐by‐group interactions were detected during cued (Figure 4C) or contextual acclimation phases (Figure 4D). However, there was a significant effect of time across trial phases reflecting normal learning in both groups (Figure 4C,D). In NOR testing, neither group showed a significant preference for the novel object at STM or LTM, and DI and absolute DI values did not differ between groups (Figure 4E,F).

**FIGURE 4:**
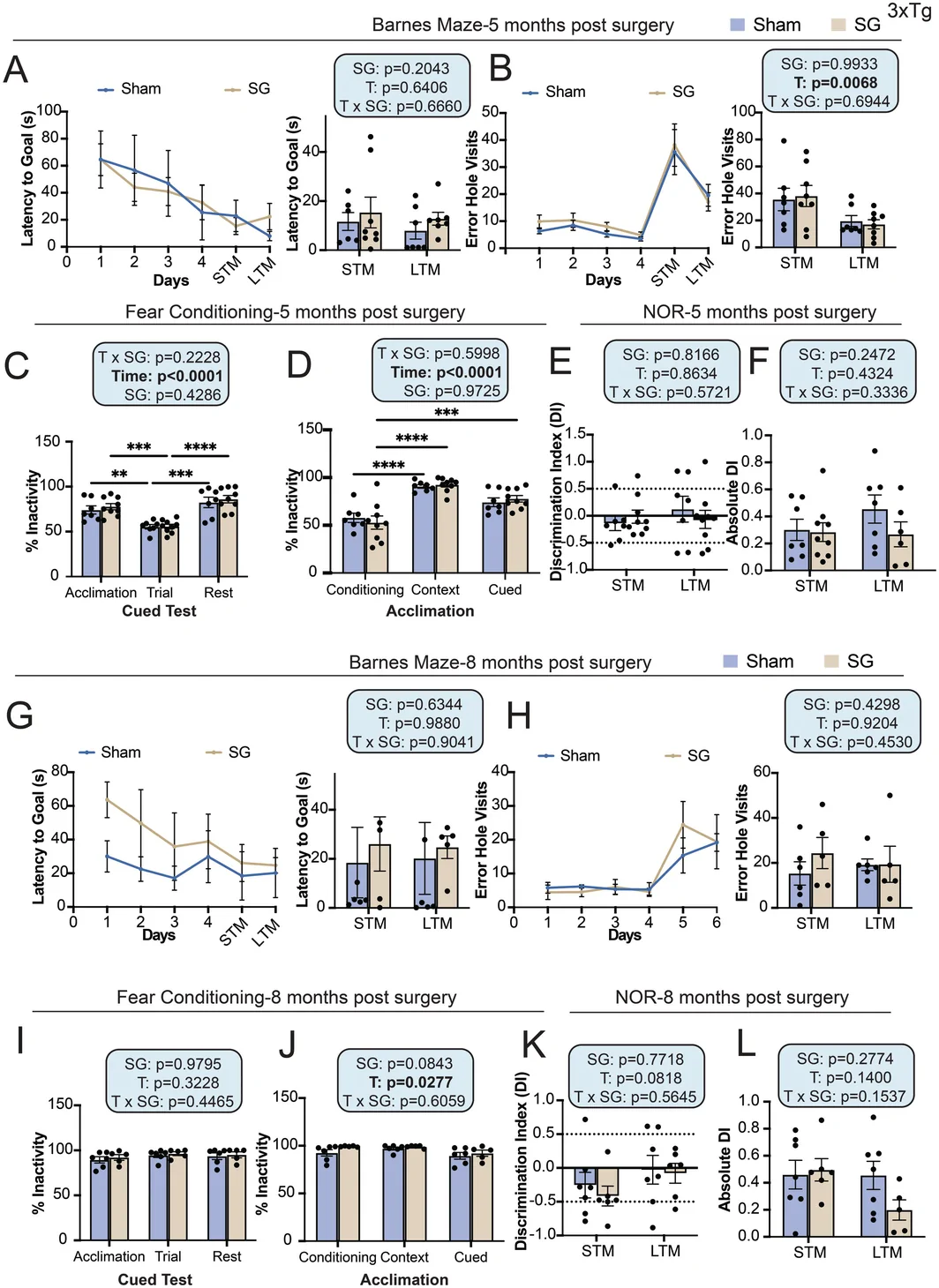
Control‐fed 3xTg‐AD mice do not exhibit cognitive deficits up to 8 months post‐surgery. (A, G) Latency of target in Barnes Maze acquisition period over the 4 days of training and in short‐term memory (STM) and long‐term memory (LTM) tests at 5 months (A) and 8 months (G) post‐surgery. (B, H) The number of error hole visits during Barnes maze training phase in STM and LTM tests at 5 months (B) and 8 months (H) post‐surgery. (C, I) Percent inactivity recorded during the cued test phase of the fear conditioning paradigm at 5 months (C) and 8 months (I) post‐surgery. (D, J) Percent inactivity recorded during the acclimation, conditioning, context, and cued phases of the fear conditioning paradigm at 5 months (D) and 8 months (J) post‐surgery. (E, K) The preference for a novel object over a familiar object was assayed at 5 months (E) and 8 months post‐surgery (K) via STM and LTM tests. The dashed lines at +0.2 and −0.2 indicate the threshold for discrimination index (DI) values showing the preference for novel or familiar objects. (F, L) Absolute DI was plotted to show the magnitude of discrimination regardless of the direction of preference at 5 months (F) and 8 months (L) post‐surgery in 3xTg‐AD mice. (A–L) Statistics for the overall effects of surgery, time, and the interaction represent the *p*‐value from a two‐way ANOVA. **p < 0.01, ***p < 0.001, ****p < 0.0001 from Sidak's multiple comparison test.

Behavioral testing repeated at 8 months post‐surgery (13 months of age) again revealed no significant group differences across any cognitive domain. BM performance at STM and LTM did not differ between SG and sham mice in latency to goal or error hole visits (Figure 4G,H). Path length analysis again confirmed no significant differences between groups across training days (Figure S5B). FC was unaffected by surgery during cued (Figure 4I) and contextual acclimation phases (Figure 4J), and NOR discrimination indices and absolute DI values at STM and LTM did not differ between groups (Figure 4K,L).

It is important to note that 3xTg Sham mice and WT mice had similar cognitive performance across all tested behavioral parameters and timepoints (Figure S6A–F). Thus, the absence of detectable cognitive differences at both timepoints most likely reflects insufficient baseline cognitive deficit in control‐fed 3xTg‐AD mice at these ages, rather than a true absence of SG effect. To address this, we examined the impact of SG on metabolism, cognition, and AD‐specific pathology under lifelong WD exposure. Long‐term WD consumption exacerbates multiple hallmarks of AD pathology, including neuroinflammation, Aβ deposition, and tau pathology in transgenic mouse models (Wiȩckowska‐Gacek et al. 2021; Graham et al. 2016; Sonsalla et al. 2025), providing a disease context in which the compound effects of genetic and dietary risk factors interact to exacerbate AD outcomes. WD represents a clinically relevant metabolic stressor which, when combined with AD‐prone genetics, can recapitulate the metabolic burden present in the human disease spectrum. We hypothesized that the combined metabolic and neuropathological burden of persistent WD feeding would unmask pro‐cognitive benefits of SG not detectable in the Control‐fed cohort.

### 2.5 SG Reduces Adiposity, Shifts Substrate Utilization, and Transiently Restores Glucose Homeostasis in 3xTg‐AD Mice Under Persistent Western Diet Conditions

A separate cohort of female 3xTg‐AD mice was preconditioned on WD for 8 weeks prior to surgery and maintained on WD throughout the 12‐month post‐surgical period (Figure 5A). Body weight, food intake, body composition, and metabolic parameters were monitored longitudinally, with glucose homeostasis assessed at 1 and 9 months post‐surgery. Consistent with the Control‐fed cohort, SG mice showed attenuated weight gain relative to shams throughout the study period (Figure 5B), driven by a significant reduction in percent fat mass at 5 and 8 months post‐surgery (Figure 5C) and a trend toward greater lean mass (Figure 5D). Food intake did not differ significantly between groups (Figure 5E), confirming that reduced caloric intake did not account for the lower adiposity observed in SG mice, regardless of dietary context. Consistent with these findings, similar reductions in body weight and fat mass were observed in WT‐WD SG mice (Figure S7A–D), confirming that the metabolic benefits of SG on body composition are independent of the AD transgenic background.

**FIGURE 5:**
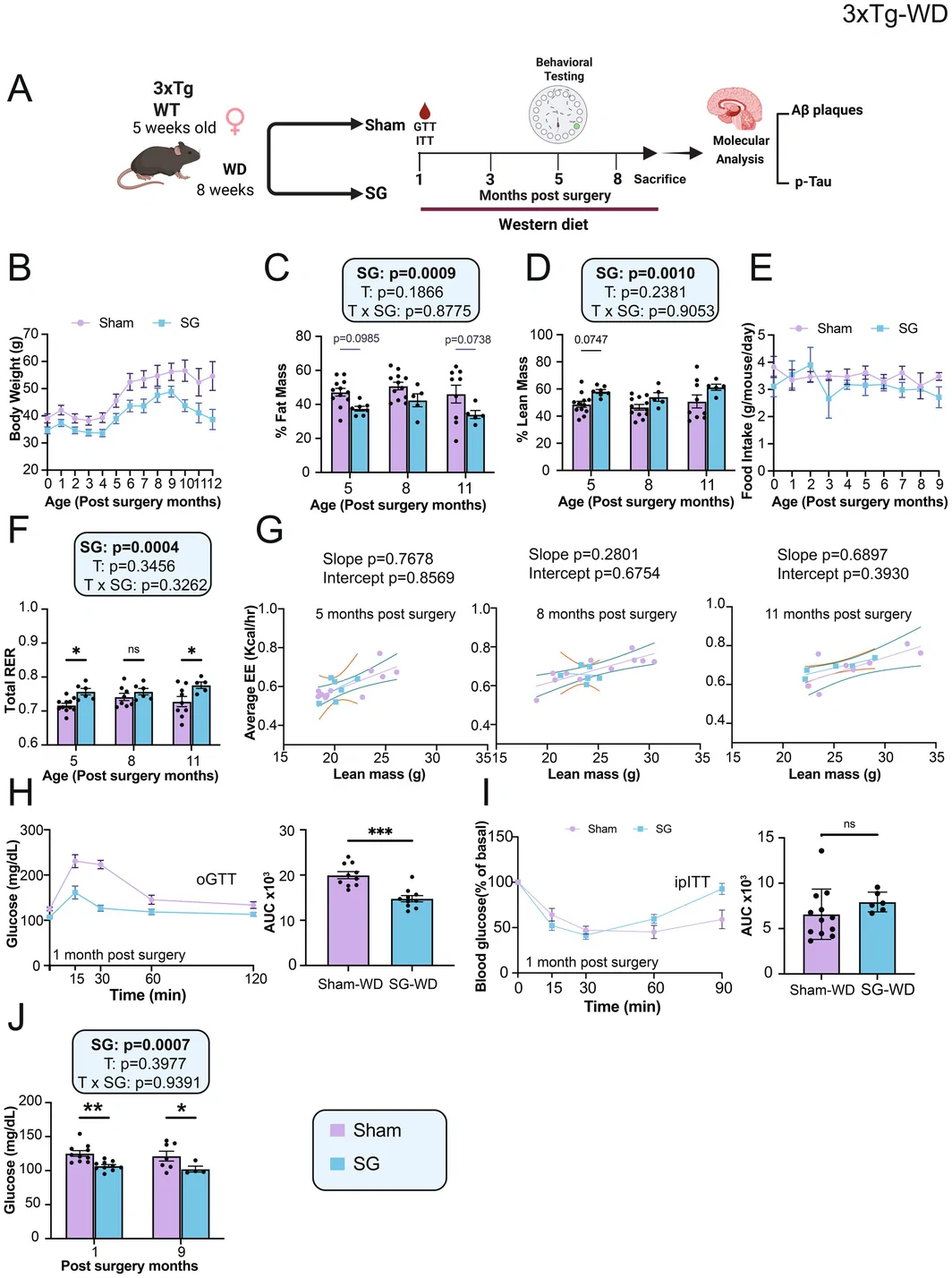
Sleeve gastrectomy reduces adiposity and improves glucose tolerance in WD‐fed 3xTg‐AD mice. (A) Experimental design of the study using female 3xTg‐AD mice. Female 3xTg‐AD and wild‐type (WT) mice were preconditioned on a Western diet (WD) for 8 weeks prior to weight‐matched randomization to receive Sham or SG followed by continued lifelong Western diet. (B) Bodyweight of female 3xTg‐AD mice longitudinally tracked 12 months post‐surgery. (C) Percent fat mass, (D) percent lean mass assessed by echoMRI at 5, 8, and 11 months post‐surgery. (E) Average daily food intake monitored over 12 months post‐surgery. (F) Average respiratory exchange ratio (RER) at 5, 8, and 11 months post‐surgery. (G) Energy expenditure (EE) normalized to lean mass at 5, 8, and 11 months post‐surgery. (H) Oral glucose tolerance test (oGTT) performed at 1‐month post‐surgery with corresponding area under the curve (AUC). (I) Intraperitoneal insulin tolerance test (ipITT) performed at 1‐month post‐surgery with corresponding AUC. (B–E). (J) Fasting blood glucose levels at 1 and 9 months post‐surgery in female 3xTg AD mice. (C, D, F, G, J) statistics show the overall effects of surgery (SG), time (T), and their interaction (T × SG) from a two‐way ANOVA; with *p < 0.05, **p < 0.01, ***p < 0.001, from Sidak's multiple comparison test. (H, I) group differences in AUC were assessed by Welch's *t*‐test; *p < 0.05, **p < 0.01, ***p < 0.001. Data represented as mean ± SEM. (A) Created with www.Biorender.com.

To assess substrate utilization, mice were placed in metabolic chambers. SG mice exhibited a significantly higher RER than shams across the study period (Figure 5F), with values consistently above 0.7, reflecting a shift toward mixed fuel substrate utilization, which was most pronounced at 5 and 11 months post‐surgery. ANCOVA with lean mass as a covariate revealed no significant differences in slopes or elevations between groups at 5, 8, or 11 months post‐surgery, indicating that EE was comparable between groups after accounting for lean mass (Figure 5G). WT‐WD mice showed no surgery‐driven differences in RER or EE (Figure S7E,F).

An oGTT performed 1‐month post‐surgery demonstrated that WD‐fed sham mice were glucose intolerant, while SG mice showed fully restored glucose tolerance (Figure 5H), indicating that SG rapidly reversed diet‐induced glucose intolerance. An ipITT at the same timepoint revealed no significant difference in insulin sensitivity between groups (Figure 5I), suggesting that the improvement in glucose tolerance occurred independently of peripheral insulin action. Notably, fasting blood glucose was significantly reduced in SG mice at both 1 and 9 months post‐surgery under persistent WD conditions (Figure 5J). In contrast, there was no difference in the AUC as measured during oGTT 1‐month post‐surgery in WT‐WD SG (Figure S7G). However, there was a trend toward a similar rapid descent to baseline serum glucose levels at 30 and 60 min; but (*p* = 0.058, p = 0.084), no differences in fasting glucose among groups (Figure S7I). There was also a strong trend toward improved insulin sensitivity in WT‐WD SG mice (*p* = 0.0541; Figure S7H). These findings suggest that the robust glucose tolerance rescue observed in 3xTg‐AD SG mice may reflect genotype‐specific metabolic adaptations.

By 9 months, however, glucose tolerance and insulin sensitivity no longer differed between groups (Figure S8A,B), driven largely by progressive normalization of glucose tolerance in the sham cohort over time.

Collectively, these data demonstrate that SG confers meaningful metabolic protection in female 3xTg‐AD mice under persistent dietary stress, reducing adiposity, shifting substrate utilization toward carbohydrate oxidation, and lowering fasting glycemia, which is broadly consistent with the metabolic benefits observed in the Control‐fed cohort, though with a distinct effect on substrate utilization not seen under standard dietary conditions.

### 2.6 SG Attenuates AD Neuropathology, Rescues Cognitive Deficits, and Reduces Frailty Progression Under Persistent Western Diet Conditions

We assessed spatial learning and memory using the Barnes Maze at 9 months post‐surgery and elected not to perform NOR or FC testing to reduce stress on the remaining animals. In contrast to the Control‐fed cohort where no group differences were detected (Figure 4), WD‐fed SG had greatly reduced latency to goal during the STM and LTM phases when compared to sham mice (Figure 6A, left). Consistent with this, SG mice demonstrated a significantly higher success rate in locating the escape box during both STM and LTM testing compared to sham‐WD controls (Figure 6A, middle). SG mice also traveled significantly less distance to reach the goal during both phases (Figure 6A, right), consistent with a more direct search route rather than a general reduction in locomotor activity. Importantly WT‐WD mice showed no cognitive impairment and near‐perfect success rates at this timepoint regardless of surgical group (Figure S9A–C), confirming that the cognitive deficits observed in sham‐WD 3xTg‐AD mice were driven by the interaction of WD and AD transgenic background rather than surgery or diet alone.

**FIGURE 6:**
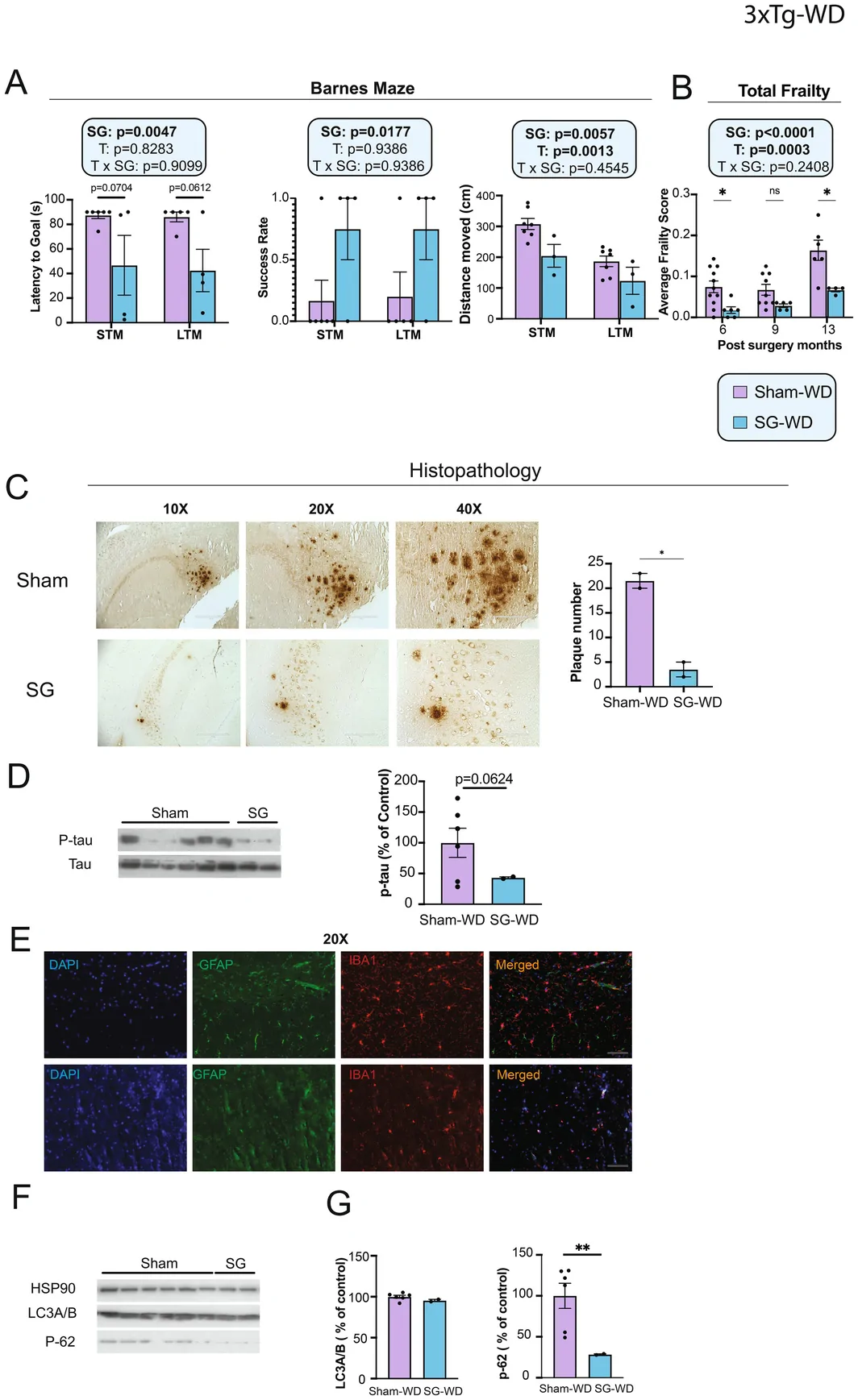
Sleeve gastrectomy rescues spatial memory deficits, reduces frailty, and improves AD‐specific pathology in Western diet‐fed 3xTg‐AD mice. (A) Latency to the target hole success rate and path length during STM and LTM probe trials of the Barnes Maze test at 9 months post‐surgery. (B) Average frailty index scores assessed longitudinally at 6, 9, and 13 months post‐surgery. (C) Representative plaque images of DAB staining with 6E10 antibody in the brains of female 3xTg‐AD 10×, 20×, and 40× magnification shown; scale bar in the 10× image is 400 μM, 20× is 200 μM, and 40× is 100 μM. Quantification of plaque number. (D) Representative western blots for phosphorylated tau (pTau Thr231) and total tau and the quantification of pTau/Tau ratio. (E) Representative immunofluorescence images of 5 μm paraffin‐embedded brain sections co‐stained for astrocytes (GFAP) and microglia (IBA1) at 20× magnification. Scale bar = 200 μm. (F) Representative immunoblots and quantification of LC3A/B and p62/SQSTM1 in brain lysates, normalized to HSP90. (G) Quantification of LC3A/B and p62 relative to expression of HSP90. For panels C, D, and G, groups were compared using Welch's *t*‐test; *p < 0.05. For panels A and B, the overall effects of surgery (SG), time (T), and their interaction (T × SG) were assessed by two‐way ANOVA; *p < 0.05. Data are represented as mean ± SEM.

Beyond cognitive outcomes, we assessed overall healthspan using a validated mouse frailty index based on the accumulation of age‐associated deficits (Whitehead et al. 2014). As expected, frailty scores increased with age in both groups (Figure 6B). Most importantly, SG mice exhibited significantly lower frailty scores than sham‐WD controls across the duration of the study (Figure 6B), and WT‐WD SG had significantly reduced frailty at 13 months (Figure S9D), confirming that the frailty benefit of SG is independent of the AD transgenic background.

The health burden of lifelong WD on this mouse cohort was reflected in the unexpectedly high mortality across both sham and SG groups through the study duration. This unfortunately substantially reduced our final group sizes. Despite this limitation, neuropathological assessment of brain tissue from all surviving mice revealed compelling differences between groups. DAB immunostaining of brain sections from 15‐month‐old, WD fed 3xTg‐AD mice showed a striking and significant reduction in plaque number in SG mice compared to sham‐WD controls (Figure 6C) consistent with the anti‐amyloidogenic effects observed in the Control‐fed cohort (Figure 2). This was accompanied by a reduction in p‐Tau levels in SG mice, though given the reduced sample size, this did not reach statistical significance (Figure 6D). Immunofluorescence co‐staining for GFAP and IBA1 demonstrated visually reduced astrocytic and microglial activation in SG mice relative to Sham‐WD controls (Figure 6E), indicating attenuation of neuroinflammation consistent with the reduced plaque burden. Formal quantification was limited by available sample numbers. We also examined autophagy pathway markers in WD‐fed mice. Immunoblotting for LC3A/B and p62 revealed that LC3A/B levels did not differ significantly between SG and sham‐WD groups (Figure 6F,G, left), while p62 was significantly reduced in SG mice (Figure 6G, right).

Taken together, these findings demonstrate that SG significantly attenuates AD neuropathology, rescues WD‐accelerated spatial memory deficits, and reduces frailty progression in 3xTg‐AD mice maintained on lifelong WD.

### 2.7 SG Does Not Improve Survival in 3xTg Animals

We completed a survival analysis for 3XTg mice in both dietary contexts. While we were largely underpowered to detect a meaningful difference in mortality between groups, there appeared to be no differences in survival between sham and SG mice in control‐fed (Figure S10A) or WD conditions (Figure S10B). However, it did appear that WD feeding accelerated mortality or at least caused an increase in morbid complications (Table S4).

## 3 Discussion

Type 2 diabetes and obesity are well‐established risk factors for AD (Silva et al. 2019). Although the precise mechanistic relationship remains incompletely understood, glucose metabolism is impaired in AD, with documented defects in glucose uptake (Duran‐Aniotz and Hetz 2016) and mitochondrial function (Baek et al. 2017; Newington et al. 2013; Krako et al. 2013). Obesity confers a nearly 2‐fold increase in AD risk relative to normal‐weight individuals (Whitmer et al. 2007), with obesity‐driven metabolic dysfunction promoting neuroinflammation and neuronal damage through mechanisms that accelerate disease progression (Valcarcel‐Ares et al. 2019; Tarantini et al. 2018; Tucsek et al. 2014). Diet further modulates AD risk, as saturated fats and refined carbohydrates, the principal components of a WD, promote excess weight gain and are associated with increased incidence of AD and cognitive dysfunction (Clemente‐Suárez et al. 2023; Hu et al. 2001; Valcarcel‐Ares et al. 2019).

Considerable effort has been directed toward understanding how targeted metabolic interventions influence AD progression. Dietary and behavioral strategies including caloric restriction (Halagappa et al. 2007; Rangan et al. 2022), protein restriction (Babygirija et al. 2024), branched‐chain amino acid restriction (Babygirija, Green, et al. 2026), and intermittent fasting (Babygirija et al. 2025; Kapogiannis et al. 2024) have each demonstrated impact on AD pathogenesis in transgenic mouse models. However, these approaches are poorly sustained in humans, where attrition rates in dietary and behavioral programs are exceedingly high (Fildes et al. 2015; Volkmar et al. 1981; Ponzo et al. 2021). Pharmacological approaches targeting the incretin axis have similarly shown mixed efficacy in preclinical AD models (Holscher 2021; Germano et al. 2024; Maskery et al. 2020) and failed to demonstrate cognitive benefit in clinical trials (Cummings et al. 2026). Furthermore, despite strong efficacy in controlled trial settings, real‐world use of GLP‐1 and GIP agonists is associated with substantially reduced effectiveness (Powell et al. 2023; Kelly et al. 2024), high therapy attrition (Miller and Brennan 2015), and weight recurrence following cessation (Horn et al. 2026; Wilding et al. 2022).

Multiple trials have established BS as the most effective intervention for obesity and obesity‐associated metabolic disease, with SG achieving rapid, durable weight loss and meaningful improvement in glycemic control and overall metabolic health (American Society for Metabolic and Bariatric Surgery 2021; Schauer et al. 2017; Heshmati et al. 2019; Mingrone et al. 2015). Critically, as a single intervention, BS confers long‐term benefits across multiple organ systems (Maciejewski et al. 2016; Syn et al. 2021; Shikora et al. 2022). Clinical evidence regarding the cognitive effects of BS, however, remains mixed. Most studies report meaningful cognitive improvements within weeks of surgery and sustained benefits through 6 months post‐SG (Tucker et al. 2020), with one recent retrospective cohort study reporting a reduced incidence of mild cognitive impairment and AD‐related dementias following surgery (Chen et al. 2025). In contrast, a large cohort study of over 51,000 subjects reported increased dementia incidence in the surgical group over a 10.5‐year follow‐up period (Kim et al. 2023).

The preclinical study of BS on AD has been limited. In a study of Roux‐en‐Y gastric bypass (alternative form of BS), male APP/PS1/TAU mice had improved cognition, downregulated Tau phosphorylation, and reduced Aβ deposition. This was associated with upregulated hippocampal GLP‐1, which is associated with improvements in AD (Mei et al. 2023). Notably, however, those animals were not obese, which limits the translational relevance to the clinical population most likely to undergo BS. To our knowledge, no prior study has examined the effects of SG specifically on AD pathology progression, which is a critical gap given the well‐established links between metabolic dysfunction and neurodegeneration. The present study addresses this gap by characterizing the effects of SG on AD‐related phenotype in a well‐validated transgenic mouse model.

The metabolic benefits of SG observed here are consistent with our prior work (Illiano et al. 2025). Under both Control and WD conditions, SG mice maintained lower body weight driven primarily by reduced fat mass accumulation, with no corresponding differences in food intake. Thus, indicating that metabolic improvements were mediated through changes in body composition rather than caloric restriction. These findings extend our previous observations of robust SG‐driven metabolic improvements in C57BL/6J mice across multiple dietary conditions (Chaudhari et al. 2021; Harris et al. 2020; Illiano et al. 2025) to a transgenic AD background, confirming that the metabolic efficacy of SG is preserved in the context of AD‐related phenotype.

Most importantly, these metabolic improvements translated to meaningful attenuation of AD neuropathology, most strikingly evidenced by a significant reduction in plaque burden selectively in the cortex. Tau phosphorylation was modulated in an epitope‐specific manner, with a trend toward reduced cortical pThr231 alongside no change in the hippocampus. This region‐specific pattern is consistent with the well‐described spatiotemporal progression of AD, in which plaque deposition first emerges in neocortical regions before spreading to the hippocampus (Selkoe 1991; Hyman et al. 1984). This preferential cortical attenuation suggests that SG may intervene at an early stage of pathological propagation, prior to hippocampal involvement or alternatively induce cortical‐plaque removal. Collectively, the reduction in cortical amyloid burden and neuroinflammation provides compelling preclinical evidence that SG is neuroprotective, and further investigation into the precise mechanisms driving these effects is warranted.

The effects of SG on tau phosphorylation were epitope‐specific and did not follow a uniform pattern. Immunofluorescence staining for pThr231 showed a trending cortical reduction in SG mice, consistent with attenuation of early GSK‐3β‐mediated tau phosphorylation (Godemann et al. 1999; Sengupta et al. 2006; Lin et al. 2007). However, AT8 (pSer202/Thr205) immunoreactivity was significantly increased in the cortex of SG mice, while pSer409 and pSer199 did not differ significantly. This divergence may reflect differential regulation of distinct tau kinase pathways. Interestingly, pThr231 is recognized as an early and clinically relevant phosphorylation event that precedes neurofibrillary tangle formation and AT8 is associated with more established tangle pathology (Jarek et al. 2024; Lin et al. 2007). The selective trending reduction in pThr231 alongside elevated AT8 may therefore suggest that SG is most effective at modulating early‐stage tau phosphorylation events, prior to the formation of mature paired helical filaments.

Under Control‐fed AD conditions, SG produced a significant improvement in peripheral insulin sensitivity, which is well‐documented following SG in both humans and mice (Chaudhari et al. 2021; Cătoi et al. 2016; Soares et al. 2023; Abu‐Gazala et al. 2018). This is particularly relevant given the well‐established role of brain insulin signaling in AD pathogenesis (Pliszka and Szablewski 2026; Liu et al. 2011). Cerebral insulin resistance impairs the PI3K/AKT/mTOR signaling axis, leading to dysregulation of autophagy and impaired clearance of Aβ and hyperphosphorylated tau (Razani et al. 2021; Ebrahim et al. 2024). Restored peripheral insulin sensitivity following SG may therefore improve central insulin signaling, promoting region‐specific modulation of autophagic flux and plaque clearance. Consistent with this interpretation, pAKT levels were inversely associated with autophagy pathway activation across brain regions. In the cortex, SG mice exhibited elevated pAKT alongside downregulation of ATG5, ATG7, and ATG16L1 indicative of inactivation of the autophagy pathway in a region where plaque burden is already reduced. In contrast, in the hippocampus SG mice showed reduced pAKT, upregulation of the same initiation markers, and decreased p62, consistent with autophagy activation, enhanced autophagic flux and active cargo clearance in a region which has greater residual pathological burden. Together, these findings are consistent with a region‐specific shift in the insulin–autophagy axis and suggest that restoration of peripheral insulin sensitivity may be a key upstream driver of the neuroprotective effects observed in this model.

Interestingly, while SG significantly elevated fasting plasma total GLP‐1 levels in WT mice, this was not observed in 3xTg‐AD mice. While GLP‐1 excursions are largely driven by food intake, which was not evaluated, this data suggests that the neuroprotective effects of SG in the transgenic AD background are not primarily driven by the GLP‐1 signaling axis. Our findings are also consistent with prior studies examining antidiabetic interventions in the 3xTg‐AD model. Previous studies utilizing GLP‐1 receptor agonists have demonstrated neuroprotective properties in preclinical AD models, including restoration of PI3K/AKT signaling, reduced neuroinflammation, improved spatial memory, and reduced AD pathology; however, these effects were variable and diet and AD model‐dependent (Urkon et al. 2025; Li et al. 2018, 2020; Wang et al. 2023). Metformin has shown particularly mixed results; while short‐term administration showed some benefit through AMPK‐mediated pathways, long‐term metformin treatment in 3xTg‐AD mice impaired cognition, raising concerns about its repurposing for AD (Cho et al. 2024; Georgiou et al. 2026). The neuroprotective effects observed in our study following SG share mechanistic similarities with these pharmacological approaches, particularly the restoration of insulin signaling and promotion of autophagic clearance. However, SG is critically distinct in that its metabolic benefits are preserved in GLP‐1 receptor knockout mice (Wilson‐Pérez et al. 2013), indicating engagement of broader metabolic reprogramming beyond GLP‐1 receptor activation alone.

A notable finding was the dissociation between neuropathological improvement and cognitive outcomes in Control‐fed 3xTg‐AD mice. Despite significant reductions in cortical plaque burden and neuroinflammation, and epitope‐specific modulation of tau phosphorylation, there were no detectable improvements in memory or cognitive performance across our testing battery at 5 or 8 months post‐surgery. However, this likely reflects insufficient baseline cognitive deficit in the sham‐operated Control‐fed cohort at these time points rather than a true absence of pro‐cognitive effect. In essence, the sham mice performed at the level of WT animals suggesting they were uncharacteristically smart, precluding detection of SG‐mediated cognitive benefits. This interpretation is consistent with the well‐documented phenotypic variability of the 3xTg‐AD model, in which the onset and severity of cognitive deficits and pathological progression vary considerably across studies and institutions (Javonillo et al. 2022; Oddo et al. 2003; Sterniczuk et al. 2010; Babygirija, Green, et al. 2026; Babygirija et al. 2025).

Under persistent dietary stress, sham animals exhibited notably worse metabolic health and accelerated cognitive decline, providing sufficient deficit range to unmask SG‐mediated cognitive benefits. The absence of cognitive improvement in the Control‐fed cohort therefore reflects a limitation of the experimental model under standard dietary conditions rather than a true absence of SG effect on brain function.

A limitation of the WD arm was reduced cohort size due to age‐associated mortality across multiple experimental groups, though final numbers remained broadly consistent with those reported in other long‐term 3xTg‐AD studies (Babygirija et al. 2024, 2025; Julien et al. 2010). Nevertheless, surviving SG mice demonstrated robust and consistent benefits across metabolic, neuropathological, cognitive, and physical health domains including improved glucose homeostasis, reduced amyloid burden, modulated tau pathology, attenuated neuroinflammation, rescued spatial memory, and reduced frailty. This collectively supports the conclusion that SG attenuates AD progression even under sustained metabolic stress. While replication in larger cohorts is necessary, these findings provide strong preclinical proof‐of‐concept for SG as a disease‐modifying intervention in obesity‐associated AD.

### 3.1 Limitations

Several limitations of the present study warrant consideration. First, we exclusively used female 3xTg‐AD mice. Inclusion of male mice and additional AD mouse models would provide broader insight into how SG‐mediated benefits generalize across sexes and genetic backgrounds. Male 3xTg‐AD mice were not included as they do not reliably develop an AD‐relevant phenotype in our hands and suffer premature mortality. This male mortality phenotype has been previously reported in commercially available 3xTg‐AD colonies and is not observed in the original LaFerla‐derived line, which most likely reflects genetic drift from a lack of routine backcrossing. Second, the tau phosphorylation analysis revealed an epitope‐specific pattern that adds complexity to interpretation. While there was a trend toward reduced pThr231, AT8 immunoreactivity was elevated in SG cortex, suggesting differential regulation. Future studies evaluating the kinase‐specific mechanisms through which SG modulates tau phosphorylation is required (Ashton et al. 2021). We note that AT8 is the most extensively validated antibody in our panel, with prior work showing no AT8 positive signal in age‐matched WT mice by IHC (Javonillo et al. 2022). A fully powered comparison of tau phosphorylation between WT and 3xTg‐AD mice was not performed in the present study and remains an important goal for future work. Third, while our autophagy analyses revealed region‐specific differences in ATG proteins, LC3A/B, and p62, we did not directly measure autophagic flux, which would be required to definitively distinguish changes in autophagy initiation from impaired downstream degradation. Future studies employing flux assays or lysosomal inhibitors would clarify the functional significance of the observed protein‐level changes. Fourth, we did not directly measure brain insulin sensitivity, and we only infer these findings from peripheral assays. Fifth, while our IHC and molecular analysis distinguished cortical and hippocampal regions broadly, we did not perform sub‐region‐specific analysis within these regions. Future studies examining discrete subregions—such as CA1, CA3, and the dentate gyrus (DG) of hippocampus, or prefrontal, entorhinal cortices would provide greater spatial resolution along with region‐specific heterogeneous responses to SG that are hidden in bulk tissue analysis. Sixth, we were largely underpowered to detect survival differences between groups. This was particularly true within the WD cohort. It is also possible that we have substantial survival bias within this cohort but it is noteworthy that many of the deaths within these animals occurred as a direct consequence of metabolic and cognitive testing performed.

In the present study, mice were euthanized at 15 months of age which limits our assessment of SG‐mediated neuroprotection at more advanced disease stages. Given the well‐documented phenotypic drift and sex‐specific variability in pathological progression in this model (Sterniczuk et al. 2010; Javonillo et al. 2022; Oddo et al. 2003; Barber et al. 2024; Dennison et al. 2021) and the accelerating effect of WD on disease timeline, future studies with extended post‐surgical follow‐up will be necessary to determine the durability of SG‐mediated neuroprotection in the face of accumulating pathology. Finally, molecular analyses were conducted on bulk tissue lysates from discrete brain regions. A more spatially resolved approach—single‐cell or region‐specific transcriptomics and proteomics—will be necessary to fully delineate the mechanistic pathways through which SG exerts neuroprotective effects across distinct cell types and neural circuits.

## 4 Conclusions

In summary, this study provides the first preclinical evidence that SG attenuates AD progression in a transgenic mouse model, with benefits spanning metabolic, neuropathological, and cognitive domains. SG reduced adiposity and improved insulin sensitivity across both dietary conditions, accompanied by region‐specific reductions in cortical amyloid plaque burden and glial activation. Cognitive benefits were observed specifically in the WD cohort, where diet‐exacerbated pathology generated sufficient behavioral impairment to unmask the pro‐cognitive potential of SG. Region‐specific modulation of autophagy machinery and insulin signaling further suggests that SG engages distinct neuroprotective mechanisms across cortical and hippocampal circuits.

Critically, these neuroprotective effects were achieved through a single surgical intervention rather than sustained dietary compliance, distinguishing SG from caloric restriction and other fasting‐based approaches that are undermined by poor long‐term adherence and weight recurrence (Fildes et al. 2015; Horn et al. 2026; Goudswaard et al. 2023). Moreover, the observed benefits are unlikely to be mediated solely through the GLP‐1 axis, given that the metabolic effects of SG are preserved in GLP‐1 receptor knockout mice (Wilson‐Pérez et al. 2013) and given the lack of robust increase in fasting GLP‐1 within the SG 3xTg cohort, indicating that SG engages broader and more durable metabolic reprogramming than GLP‐1 receptor agonism alone. This mechanistic breadth may underlie the efficacy of SG in a context where pharmacological GLP‐1‐based therapies have failed to demonstrate cognitive benefit in trial (Cummings et al. 2026). Collectively, these findings support SG as a candidate disease‐modifying intervention for patients with obesity and diet‐induced metabolic dysfunction prone to AD. Determining the mechanisms through which SG modulates AD risk may inform the development of novel therapeutic strategies for AD prevention.

## 5 Materials and Methods

### 5.1 Animals

Female homozygous 3xTg‐AD mice were obtained from The Jackson Laboratory (Bar Harbor, ME, USA) and were bred and maintained at the vivarium with food and water available ad libitum. At 5 weeks of age, they were preconditioned on a high‐fat WD (Envigo, TD.88137; 42% calories from fat; 15% calories from protein; 43% calories from carbohydrates) for 8 weeks to induce obesity and glucose intolerance. Male mice were not used during this study and thus the impact of sex was not studied. Mice were housed in groups of two and had ad libitum access to food and water in a climate‐controlled environment with a 12‐h light/dark cycle. All procedures were approved by the Institutional Animal Care and Use Committee (UW‐Madison and William S. Middleton Memorial Veteran's Hospital), and animals were cared for according to guidelines set forth by the American Association for Laboratory Animal Science in an AAALAC‐accredited facility.

### 5.2 Diets

Following 8‐week obesogenic preconditioning, mice were placed in two separate dietary cohorts. In the control‐fed cohort, mice received a balanced diet (TD.180161; 21% kcal from protein, 20% from fat, 58.8% from carbohydrates) following surgery throughout the 12‐month post‐surgical period. In the WD cohort, mice were continued on WD (Envigo TD.88137; 42% kcal from fat, 15% from protein, 43% from carbohydrates; sucrose as primary carbohydrate source) throughout the 12‐month post‐surgical period. WD has been previously shown to accelerate AD progression in 3xTg‐AD and other transgenic mouse models (Wiȩckowska‐Gacek et al. 2021; Graham et al. 2016; Sonsalla et al. 2025). Detailed diet compositions are provided in Table S1.

### 5.3 Sleeve Gastrectomy and Sham Procedures

At 12 weeks of age, mice were weight matched and randomized into either a Sham or SG surgical group. In brief, mice were placed on a recovery gel diet from 48 h prior to surgery to 6 days post‐surgery before going on their respective diets (Clear H20, Westbrook, ME). At surgery, mice were anesthetized with isoflurane and received weight‐based saline, Norocillin, and buprenorphine (Ethiqua XR; Fidelis Animal Health, North Brunswick, NJ). SG consisted of a midline laparotomy, short vessel ligation, and removal of roughly 75% of the stomach and the entirety of the non‐glandular stomach using a linear cutting stapler (Medtronic, Minneapolis, MN) as previously described (Illiano et al. 2025; Chaudhari et al. 2021; Harris et al. 2020). Sham surgery similarly consisted of a laparotomy, short vessel ligation, and manipulation of the stomach along the would‐be staple line.

### 5.4 Glucose and Insulin Tolerance Tests

OGTT and ITT were performed after a 4‐h fast (7 a.m.–11 a.m.). Mice received an oral bolus of 30% glucose (1 g/kg) and blood glucose was measured at time 0, 15, 30, 60, and 120 min. For ITT mice received an intraperitoneal bolus of regular insulin (0.5 U/kg; Humulin R: Eli Lilly USA). Blood glucose was measured 0, 15, 30, 60, and 90 min. The blood glucose measurements were collected using a Bayer Contour glucose meter and test strips.

### 5.5 Body Composition and Indirect Calorimetry Analysis

Body composition was assessed using the EchoMRI Body Composition Analyzer. Indirect calorimetry was performed using Oxymax/CLAMs metabolic chamber system as previously described (Müller et al. 2021). Briefly, mice were individually housed in CLAMs cages at 22°C and measurements for CO_2_ production, O_2_ consumption, heat production, RER, and food intake were recorded for each mouse. Mice were placed in CLAMs cages for ∼48 h with a 12‐h light and dark cycle. The first 24 h were considered the acclimation period and were discarded from the analysis. Mice had access to food and water ad libitum.

### 5.6 Behavioral Assays

In the Control‐fed cohort, behavioral phenotyping was performed at 5 and 8 months post‐surgery (9 and 13 months of age, respectively). In the WD cohort, assays were performed at 9 months post‐surgery. The Novel object recognition test (NOR) was performed in an open field where the movements of the mouse were recorded via a camera that is mounted above the field. Before each test, mice were acclimatized in the behavioral room for 30 min and were given a 5‐min habituation trial with no objects on the field. This was followed by test phases that consisted of two trials that were 24 h apart: Short term memory test (STM) and Long‐term memory test (LTM). In the first trial, the mice were allowed to explore two identical objects placed diagonally on opposite corners of the field for 5 min. Following an hour after the acquisition phase, STM was performed and 24 h later, LTM was done by replacing one of the identical objects with a novel object. The results were quantified using a DI, representing the duration of exploration for the novel object compared to the old object.

For Barnes maze, the test involves three phases: habituation, acquisition training and the memory test. During habituation, mice were placed in the arena and allowed to freely explore the escape hole, escape box, and the adjacent area for 2 min. Following that during acquisition training the mice were given 180 s to find the escape hole, and if they failed to enter the escape box within that time, they were led to the escape hole. After 4 days of training, on the 5th day (STM) and 12th day (LTM) the mice were given 90 s memory probe trials. The latency to enter the escape hole, distance traveled, and average speed were analyzed using Ethovision XT (Noldus).

Associative fear memory was assessed using a standard cued and contextual fear conditioning paradigm. On the conditioning day, mice were placed in the fear‐conditioning chamber (26 × 26 × 24 cm) and allowed an 8‐min habituation period, during which baseline freezing was recorded. Mice then received three 2‐s, 0.5 mA foot shocks, each along with a 30‐s auditory cue (conditioned stimulus, CS), administered at 90‐s intervals. Contextual fear memory was assessed 24 h after conditioning by returning mice to the identical chamber for 5 min in the absence of the CS, and percent time freezing was recorded. Cued fear memory was assessed in a novel context 24 h after conditioning: mice were first allowed a 3‐min acclimation period in the novel chamber, followed by a 3‐min CS presentation, and percent freezing during each phase was recorded. All behavioral recordings were analyzed using Ethovision XT (Noldus).

### 5.7 Frailty Measurements

Frailty was assessed longitudinally using a 28‐item frailty index based on the accumulation of age‐associated deficits, as described by Whitehead et al. (2014). Items assessed include alopecia, coat condition, kyphosis, gait disorders, body condition score, ocular changes, breathing rate, and other clinical signs, each scored as 0 (absent), 0.5 (mild), or 1 (severe deficit). The frailty index was calculated as the sum of all deficit scores divided by the total number of items assessed.

### 5.8 Immunoblotting

Tissue samples from the brain regions were lysed in cold RIPA buffer supplemented with phosphatase inhibitor and protease inhibitor cocktail tablets (Thermo Fisher Scientific, Waltham, MA, USA) using a FastPrep 24 (M.P. Biomedicals, Santa Ana, CA, USA) with bead‐beating tubes (16466‐042) from (VWR, Radnor, PA, USA) and zirconium ceramic oxide bulk beads from (Thermo Fisher Scientific, Waltham, MA, USA). Protein lysates were then centrifuged at 13,300 rpm for 10 min and the supernatant was collected. Protein concentration was determined by Bradford (Pierce Biotechnology, Waltham, MA, USA). 20 μg protein was separated by SDS–PAGE (sodium dodecyl sulfate–polyacrylamide gel electrophoresis) on 8%, 10%, or 16% resolving gels (ThermoFisher Scientific, Waltham, MA, USA) and transferred to PVDF membrane (EMD Millipore, Burlington, MA, USA). Autophagy markers including autophagy proteins ATG5, ATG7, and ATG16L1, as well as autophagosome formation marker light chain 3A/B (LC3A/B), and the autophagy receptor p62 (sequestosome 1, SQSTM1) were immunoblotted (Babygirija et al. 2024, 2025). Tau pathology was assessed by immunoblotting for a panel of phosphorylated tau epitopes, pThr231, AT8 (pSer202/Thr205), pSer409, and pSer199, normalized to total tau (HT7), which specifically detects human tau and is appropriate for the 3xTg‐AD model expressing human tau transgene (tauP301L). Antibody vendors, catalog numbers and the dilution used are provided in Table S3. Imaging was performed using an Image Quant 800 (Amersham). Quantification was performed by densitometry using NIH ImageJ software.

### 5.9 Histology for AD Neuropathology Markers

Mice were euthanized by cervical dislocation after 3 h fast, and the right hemisphere was fixed in formalin for histology whereas the left hemisphere was snap‐frozen for biochemical analysis. Two non‐adjacent tissue sections per animal were collected from microdissected cortical and hippocampal regions. For amyloid plaque staining, briefly, brain sections were deparaffinized and rehydrated according to standard protocol. For epitope retrieval, mounted slides were pretreated in 70% formic acid at room temperature for 10 min. Tissue sections were subsequently blocked with normal goat serum (NGS) at room temperature for 1 h, then incubated with monoclonal antibodies 6E10 (1:100) at 4°C overnight. Aβ immunostained profiles were visualized using diaminobenzidine chromagen. For p‐Tau staining and glial activation, brains were analyzed with p‐Tau Thr231, anti‐GFAP (astrocytic marker), and anti‐Iba1 (microglial marker) antibodies respectively. The following primary antibodies were used: phospho‐Tau (Thr231) monoclonal antibody (AT180) (Thermo Fisher Scientific; #MN1040, 1:100), anti‐GFAP (Thermo Fisher; #PIMA512023; 1:1000), anti‐IBA1 (Abcam; #ab178847; 1:1000). Sections were imaged using an EVOS microscope (Thermo Fisher Scientific Inc., Waltham, MA, USA) at a magnification of 10×, 20×, and 40×. ImageJ was used for quantification by converting images into binary images via an intensity threshold and positive area was quantified (Rigby et al. 2021). For each section, the entire tissue area was imaged and quantified uniformly across all animals. Plaque number, percent area, and glial immunoreactivity were averaged across the two sections per animal.

### 5.10 GLP‐1 Enzyme Immunoassay

Total plasma GLP‐1 levels were measured using the GLP‐1 EIA Kit (Sigma‐Aldrich, Cat #RAB0201) according to the manufacturer's instructions. Plasma samples were collected after a 4‐h fast and stored at −80°C until analysis. Concentrations were calculated from a standard curve ranging from 0.1–1000 pg/mL.

### 5.11 Statistical Analysis

All statistical analyses were conducted using Prism, version 11.0.0 (GraphPad Software Inc., San Diego, CA, USA). Comparisons between two independent groups were done using Welch's *t*‐test and tests involving multiple factors were analyzed by either a two‐way analysis of variance (ANOVA) with surgery, time or brain region as variables or by one‐way ANOVA, followed by a Sidak's post hoc test as specified in the figure legends. Mann–Whitney U tests were additionally performed as a nonparametric complement to select two‐way ANOVA analyses, with results summarized in Table S2. Alpha was set at 5% (*p* < 0.05 considered to be significant). Data are presented as the mean ± SEM unless otherwise specified.

## Author Contributions

R.B., J.A.I., D.W.L., and D.A.H. conceived of and designed the experiments. R.B., J.A.I., M.M.S., G.Z., C.R.J, T.M., M.P., C.W., A.R.K.N., F.X., J.M.W., D.W.L., and D.A.H. analyzed the data. R.B. and D.A.H. wrote the manuscript.

## Funding

This work was supported by the National Institute on Aging (R03AG088813, AG056771, AG081482, AG084156, AG085898, AG094153, P30 AG092586, P30‐AG062715), National Institute of Diabetes and Digestive and Kidney Diseases (DK125859), Wisconsin Partnership Program (ID 6770‐2024), Wisconsin Alumni Research Foundation (UW Distinguished Research Fellow, Vilas Faculty Early Career Investigator Award), and US Department of Veterans Affairs (I01‐BX004031, IS1‐BX005524).

## Conflicts of Interest

D.W.L. has received funding from, and is a scientific advisory board member of, Aeovian Pharmaceuticals, which seeks to develop novel, selective mTOR inhibitors for the treatment of various diseases. The other authors declare no conflicts of interest.

## Supporting information


**Figure S1:** Sleeve gastrectomy reduces adiposity and improves insulin sensitivity independent of food intake in Control‐fed WT mice. (A) Body weight of female WT mice longitudinally tracked over 12 months post‐surgery. (B) Percent fat mass, (C) percent lean mass assessed by echoMRI at 6 months post‐surgery. (D) Average daily food intake monitored over 12 months post‐surgery. (E) Energy expenditure (EE) normalized to lean mass at 6 months post‐surgery shown with ANCOVA‐derived slope and intercept *p*‐values. (F) Respiratory exchange ratio (RER) during light and dark phases at 6 months post‐surgery. (G) Oral glucose tolerance test (oGTT) performed at 2 months post‐surgery with corresponding area under the curve (AUC). (H) Intraperitoneal insulin tolerance test (ipITT) performed at 2 months post‐surgery with corresponding AUC. (I) Plasma total cholesterol and (J) triglycerides measured at endpoint in WT Sham and SG mice. (B, C, G) The overall effects of surgery (SG), time (T), and their interaction (T × SG) from two‐way ANOVA; *p < 0.05, **p < 0.01 from Sidak's multiple comparisons test. (G–J) Group differences in AUC were assessed by Welch's *t*‐test. (A–I) *n* = 10–12/group and (J) *n* = 5–7/group. Data represented as mean ± SEM.
**Figure S2:** Energy balance in Control‐fed 3xTg‐AD mice 10 months post‐surgery. (A) Respiratory exchange ratio (RER) and energy expenditure (EE) during light and dark phases at 10 months post‐surgery in Sham and SG female 3xTg‐AD mice. Statistics reflect the overall effects of surgery (SG), time (T), and their interaction (T × SG) from a two‐way ANOVA. Data are represented as mean ± SEM.
**Figure S3:** SG elevates fasting plasma GLP‐1 levels in WT but not 3xTg‐AD mice. (A) Total fasting plasma GLP‐1 levels (pg/mL) measured by ELISA in WT and 3xTg‐AD Sham and SG mice from the control‐fed cohort. Statistics reflect the overall effects of surgery (SG), Genotype (GDdT), and their interaction (T × SG) from a two‐way ANOVA. *p < 0.05. *n* = 5–6/group. Data represented as mean ± SEM.
**Figure S4:** SG differentially modulates tau phosphorylation across multiple epitopes and brain regions in control‐fed 3xTg‐AD mice. (A) Representative western blot images showing pTau Thr231, AT8 (pSer202/Thr205), pSer409, pSer199, and total Tau from cortical and hippocampal lysates of 15‐month‐old female 3xTg‐AD mice. Samples were randomized across lanes to allow blinded quantification. Total tau was used as loading control for normalization of all pTau signals. (B) Quantification of pTau Thr231/total tau ratio in cortex and hippocampus. (C) Quantification of AT8 (pSer202/Thr205)/total tau ratio in cortex and hippocampus. (D) Quantification of pSer409/total tau ratio in cortex and hippocampus. (E) Quantification of pSer199/total tau ratio in cortex and hippocampus. (F) Representative immunofluorescence images of pTau Thr231 (green), DAPI (blue), and merged channels in cortical and hippocampal sections at 20× magnification. Scale bar = 200 μm. Quantification of pTau Thr231 IF intensity (intensity/pixel) in cortex and hippocampus. Statistics show overall effects of surgery (SG), region (R), and their interaction (*R* × SG) from two‐way ANOVA. Mann–Whitney *U* tests were additionally performed within each brain region; results summarized in Table S2. *N* = 5–6/group. Data represented as mean ± SEM.
**Figure S5:** There were no differences in BM pathlength in control‐fed 3xTg‐AD mice. (A) Distance moved (cm) during Barnes Maze training (Days 1–4) and probe trials (STM and LTM) at 5 months post‐surgery (9 months of age) in Sham and SG 3xTg‐AD mice. (B) Distance moved (cm) during Barnes Maze training (Days 1–4) and probe trials (STM and LTM) at 8 months post‐surgery (13 months of age). Statistics for the overall effects of surgery, time and the interaction represent the *p*‐value from a 2‐way ANOVA. * *p* < 0.05 **p < 0.01, ****p < 0.0001 from Sidak's multiple comparison test. *N* = 8–10/group. Data represented as mean ± SEM.
**Figure S6:** Control‐fed WT mice do not exhibit cognitive deficits. (A) Latency to goal (s) during Barnes Maze (BM) training phase and STM and LTM tests in WT mice at 7 months of age. (B) Error hole visits during BM training phase and STM and LTM tests in WT mice at 7 months of age. (C) Distance moved (cm) during BM training phase and STM and LTM tests in WT mice at 7 months of age. (D) Percent inactivity recorded during the cued test phase of the fear conditioning paradigm in WT mice at 7 months of age. (E) Percent inactivity recorded during the acclimation, context, and cued phases of the fear conditioning paradigm in WT mice at 7 months of age. (F) Discrimination index (DI) during STM and LTM Novel Object Recognition testing in WT mice at 7 months of age. Dashed lines at +0.2 and −0.2 indicate the threshold for novel or familiar object preference. (A–F) Statistics show the overall effects of surgery (SG), time (T), and their interaction (T × SG) from two‐way ANOVA. *p < 0.05, **p < 0.01, ****p < 0.0001 from Sidak's multiple comparison test. Data represented as mean ± SEM. *n* = 12 Sham, *n* = 11 SG.
**Figure S7:** Sleeve gastrectomy reduces adiposity without altering energy balance in WT mice under persistent western diet conditions. (A) Body weight (g) of female WT mice tracked longitudinally over 12 months post‐surgery. (B) Percent fat mass and (C) percent lean mass assessed by body composition analysis at 5, 8, and 11 months post‐surgery. (D) Average daily food intake monitored over 12 months post‐surgery. (E) Total respiratory exchange ratio (RER) at 5, 8, and 11 months post‐surgery. (F) Energy expenditure (EE) normalized to lean mass with ANCOVA‐derived slope and intercept *p*‐values shown for each timepoint confirming no significant surgery effect after lean mass correction. (G) Oral glucose tolerance test (oGTT) performed at 1‐month post‐surgery with corresponding area under the curve (AUC). (H) Intraperitoneal insulin tolerance test (ipITT) performed at 1‐month post‐surgery with corresponding AUC. (I) Fasting blood glucose at 1 and 9 months post‐surgery. (B–C, E, I) Statistics show overall effects of surgery (SG), time (T), and their interaction (T × SG) from two‐way ANOVA; *p < 0.05, **p < 0.01, ***p < 0.001 from Sidak's multiple comparison test. (G–H) Group differences in AUC assessed by Welch's *t*‐test. Data represented as mean ± SEM.
**Figure S8:** Glucose homeostasis is comparable between SG and Sham mice at 9 months post‐surgery in WD‐fed 3xTg‐AD mice. (A) Oral glucose tolerance test (oGTT) and corresponding area under the curve (AUC) at 9 months post‐surgery in Sham‐WD and SG‐WD female 3xTg‐AD mice. (B) Intraperitoneal insulin tolerance test (ipITT) and corresponding AUC at 9 months post‐surgery. ns = not significant. Data are represented as mean ± SEM.
**Figure S9:** WT‐WD mice do not exhibit cognitive impairment but have reduced frailty following SG. (A) Latency to goal (s) during STM and LTM Barnes Maze testing in WT‐WD Sham and SG mice. (B) Success rate during STM and LTM Barnes Maze testing in WT‐WD Sham and SG mice. (C) Path length during STM and LTM BM testing in WT‐WD Sham and SG mice. (D) Average frailty score assessed at 6, 9, and 13 months post‐surgery in WT‐WD Sham and SG mice. (A, B) Group differences assessed by Welch's *t*‐test. (C) Statistics show overall effects of surgery (SG), time (T), and their interaction (T × SG) from two‐way ANOVA; **p < 0.01 from Sidak's multiple comparison test at 13 months post‐surgery. Data represented as mean ± SEM.
**Figure S10:** Survival probability and cause of death in 3xTg‐AD mice under lifelong western diet feeding. (A) Kaplan–Meier survival curve showing probability of survival over time (days) between Sham and SG animals under control‐diet conditions. (B) Kaplan–Meier survival curve showing probability of survival over time (days) between Sham and SG animals under WD conditions.

**Table S1:** Diet composition of control and Western diets used in this study.
**Table S2:** Mann–Whitney *U* test results comparing SG versus sham within each brain region.
**Table S3:** Antibodies used for both western blotting and immunohistochemistry.
**Table S4:** Individual cause of death in control‐fed (A) and WD (B) cohorts.

## Data Availability

The data that support the findings of this study are available from the corresponding author upon reasonable request.
