# Supplementary material for: Sleeve Gastrectomy Improves Metabolic Health, Cognition, and Alzheimer's Disease Pathology in 3xTG Mice
Source: Aging Cell. 2026 Oct 13;25(10):e70767. doi: 10.1111/acel.70767 (PMC13660725; doi:10.1111/acel.70767)

Figure S1

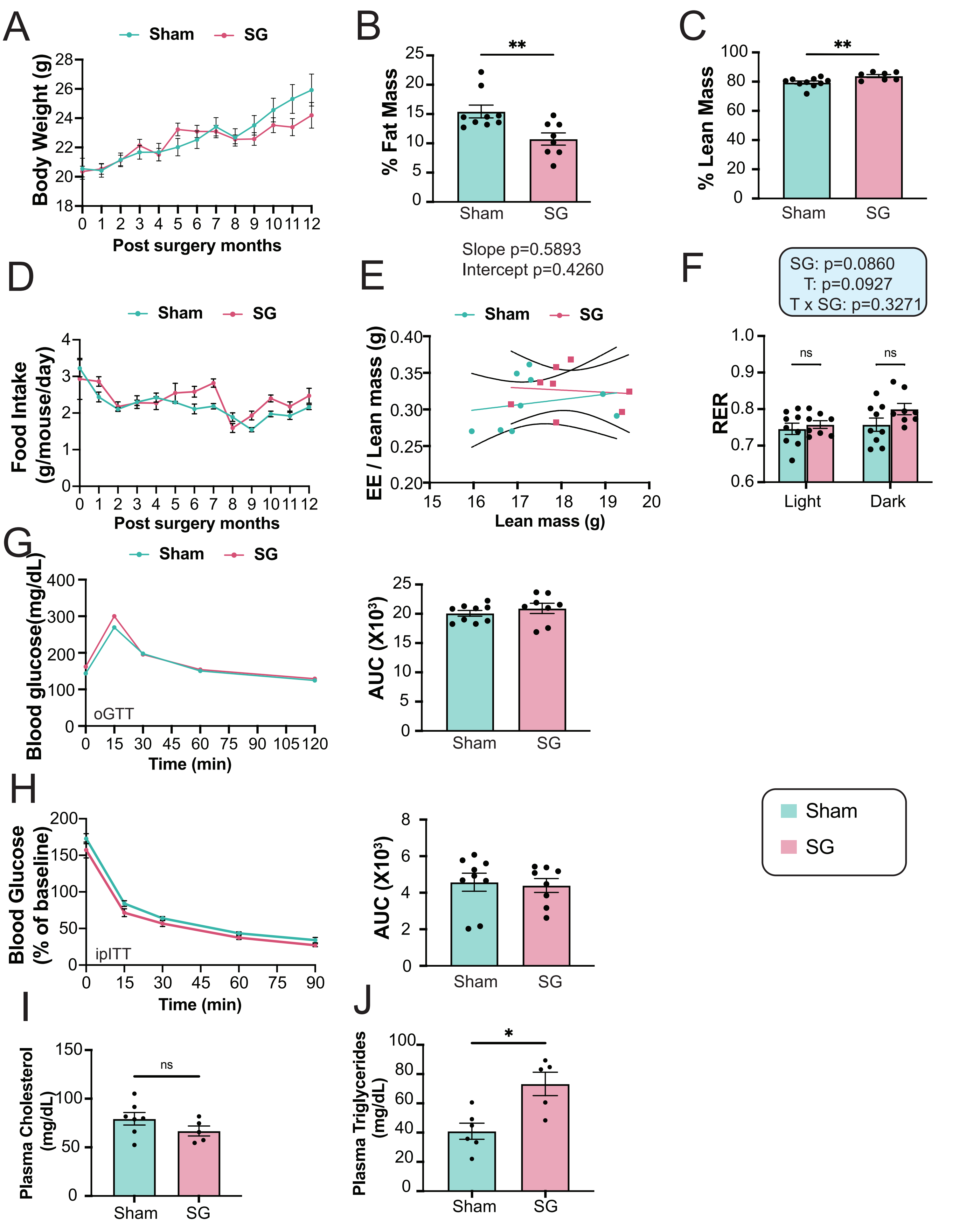
WT
A
Sham
SG
Body Weight (g)
Post surgery months
B
% Fat Mass
**
Sham
SG
C
% Lean Mass
**
Sham
SG
D
Food Intake (g/mouse/day)
Post surgery months
E
Slope p=0.5893
Intercept p=0.4260
EE / Lean mass (g)
Lean mass (g)
F
SG: p=0.0860
T: p=0.0927
T x SG: p=0.3271
RER
ns
ns
Light
Dark
G
Blood glucose(mg/dL)
oGTT
Time (min)
AUC (X10³)
H
Blood Glucose (% of baseline)
ipITT
Time (min)
AUC (X10³)
I
Plasma Cholesterol (mg/dL)
ns
J
Plasma Triglycerides (mg/dL)
*

Figure S2

3xTg

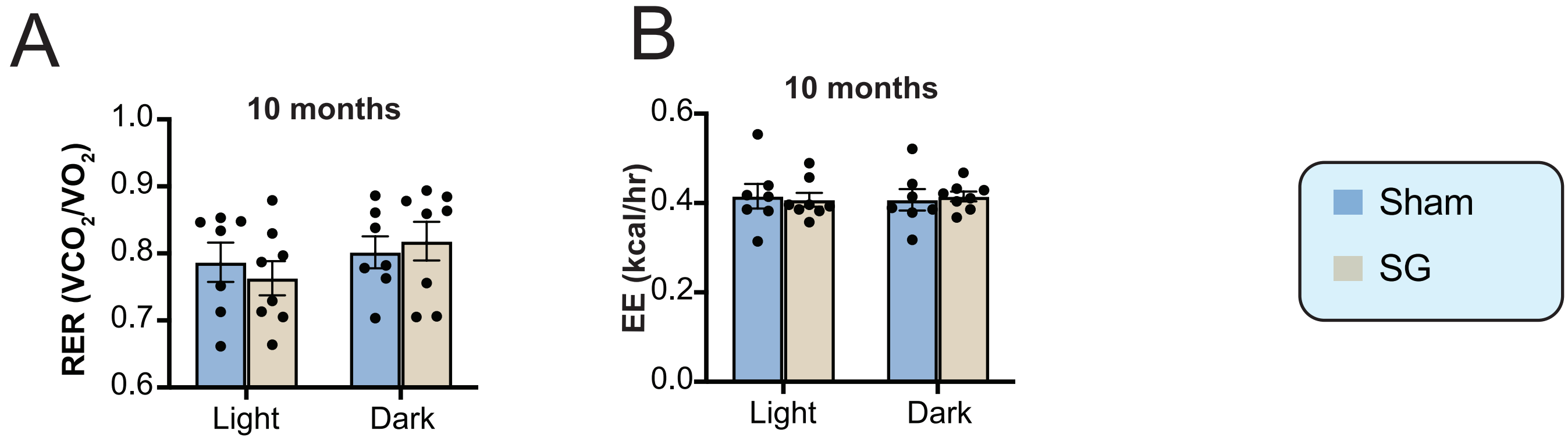

Figure S3

A

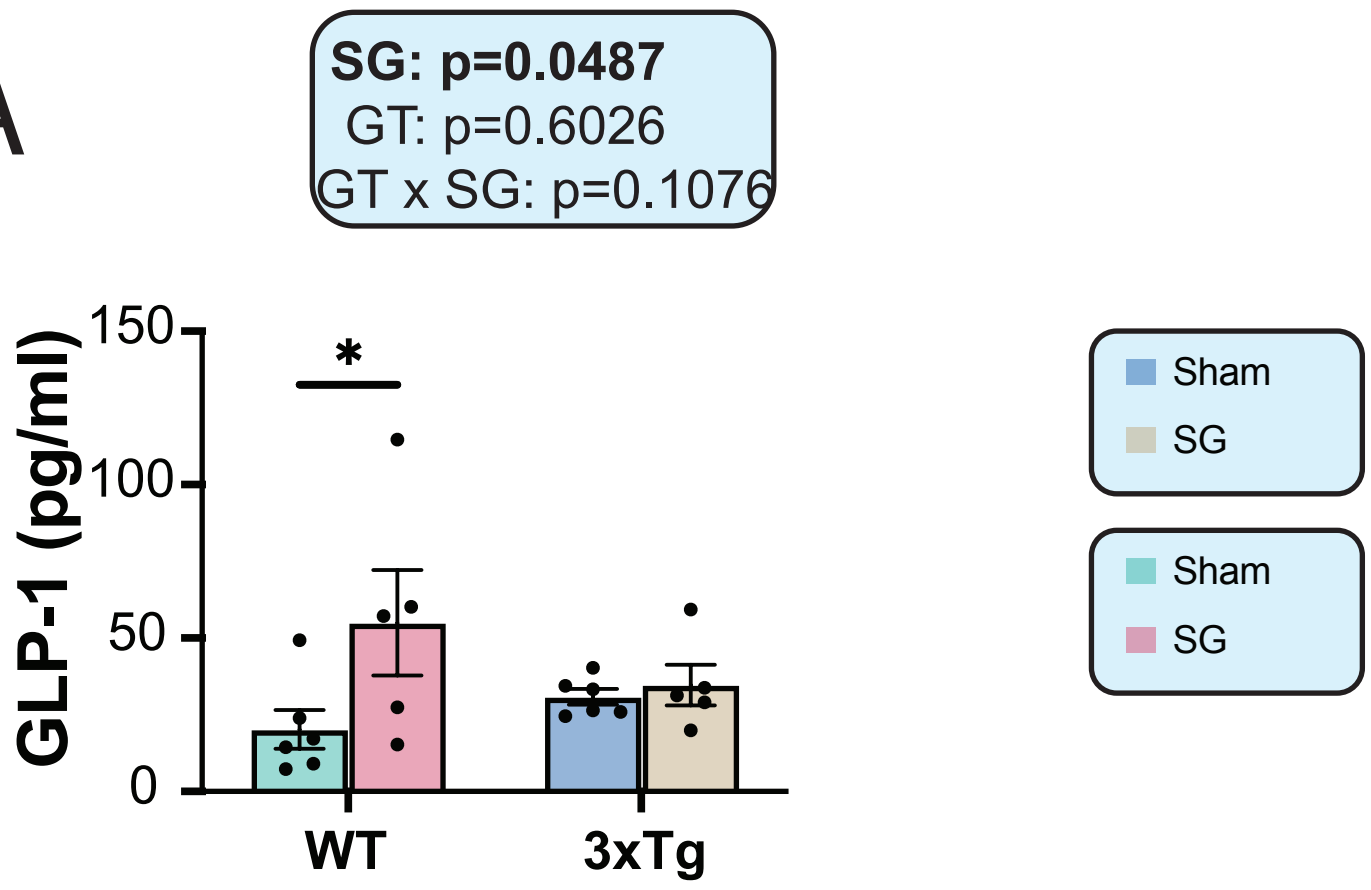
SG: p=0.0487
GT: p=0.6026
GT x SG: p=0.1076
GLP-1 (pg/ml)
150
100
50
0
*
WT
3xTg
Sham
SG
Sham
SG

Figure S4

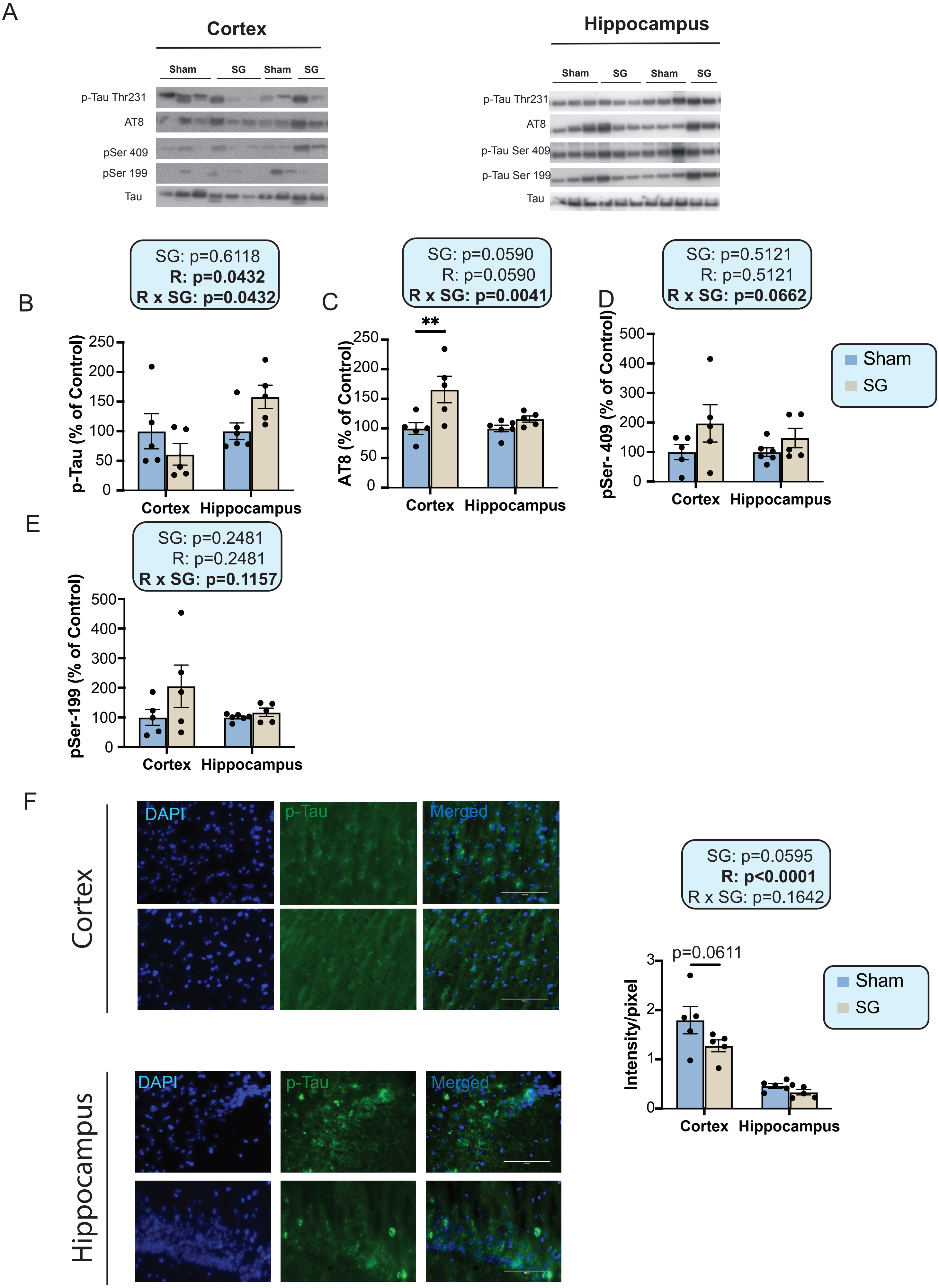

A
Cortex
Sham
SG
Sham
SG
p-Tau Thr231
AT8
pSer 409
pSer 199
Tau
Hippocampus
Sham
SG
Sham
SG
p-Tau Thr231
AT8
p-Tau Ser 409
p-Tau Ser 199
Tau
B
SG: p=0.6118
R: p=0.0432
R x SG: p=0.0432
p-Tau (% of Control)
Cortex
Hippocampus
C
SG: p=0.0590
R: p=0.0590
R x SG: p=0.0041
**
AT8 (% of Control)
Cortex
Hippocampus
D
SG: p=0.5121
R: p=0.5121
R x SG: p=0.0662
pSer- 409 (% of Control)
Cortex
Hippocampus
Sham
SG
E
SG: p=0.2481
R: p=0.2481
R x SG: p=0.1157
pSer-199 (% of Control)
Cortex
Hippocampus
F
Cortex
DAPI
p-Tau
Merged
Hippocampus
DAPI
p-Tau
Merged
SG: p=0.0595
R: p<0.0001
R x SG: p=0.1642
p=0.0611
Intensity/pixel
Sham
SG
Cortex
Hippocampus

Figure S5

3xTg

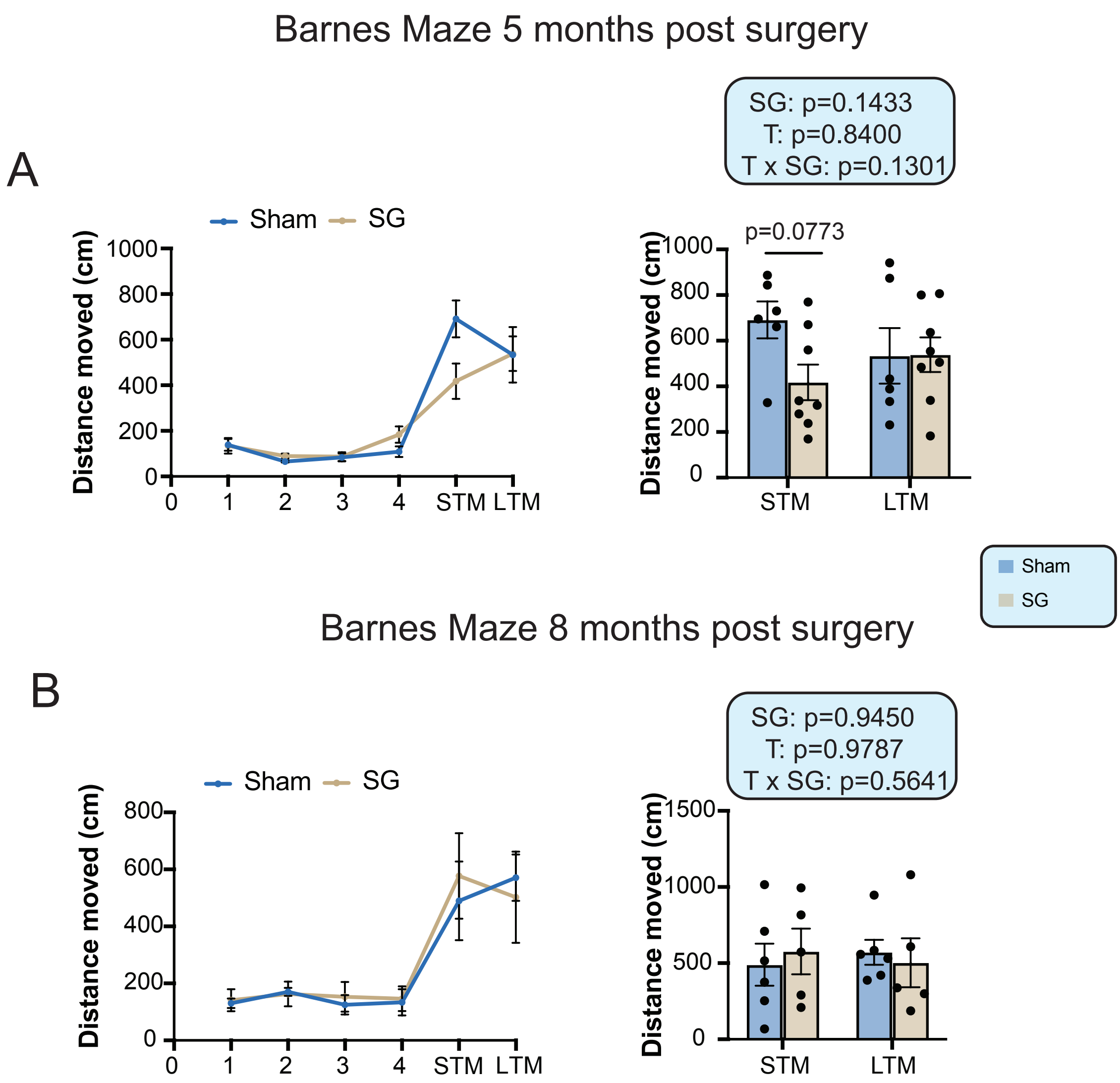

Figure S6

WT

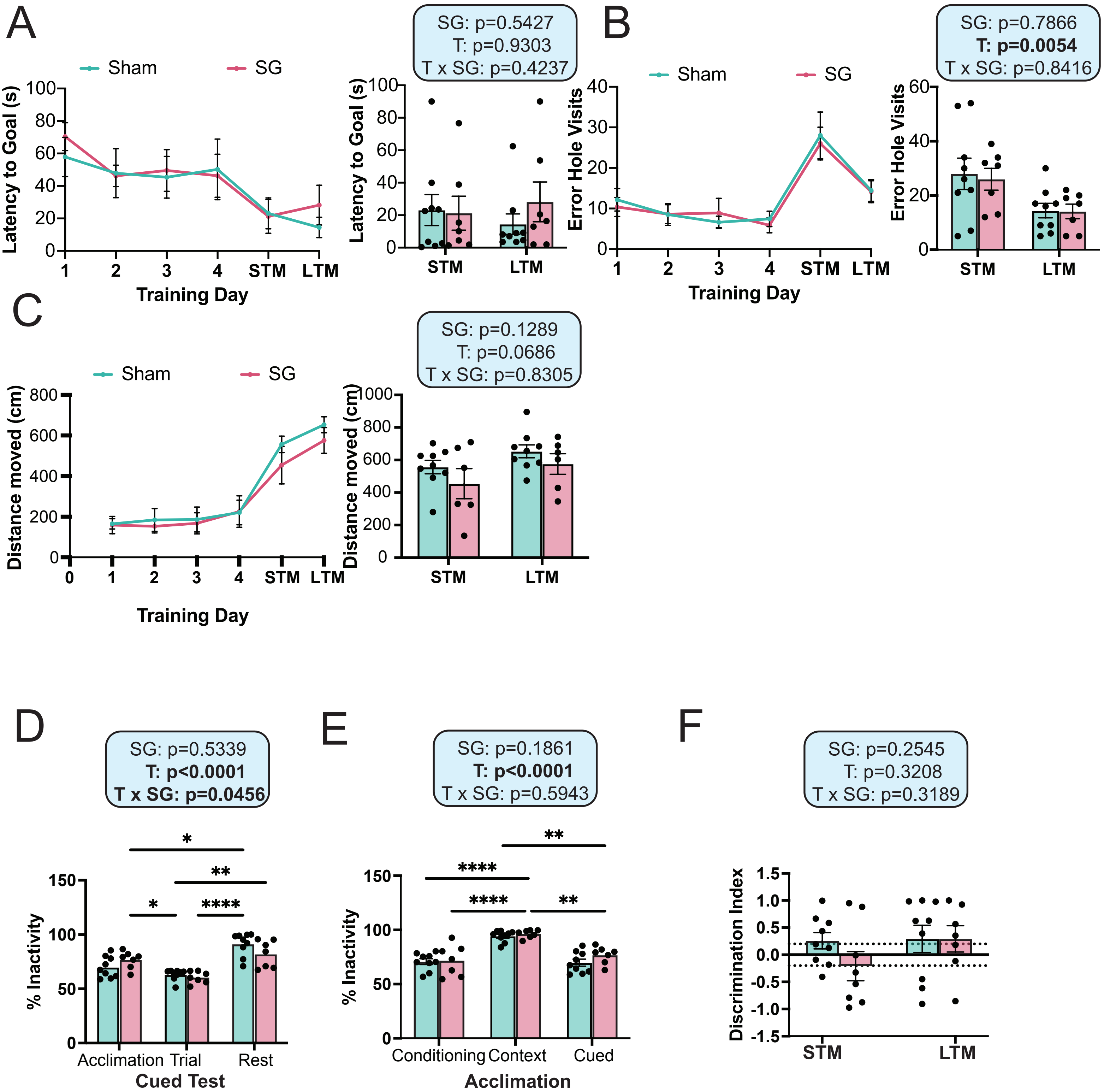

A
Sham
SG
Latency to Goal (s)
Training Day
1 2 3 4 STM LTM
SG: p=0.5427
T: p=0.9303
T x SG: p=0.4237
STM LTM
B
Error Hole Visits
SG: p=0.7866
T: p=0.0054
T x SG: p=0.8416
C
Distance moved (cm)
SG: p=0.1289
T: p=0.0686
T x SG: p=0.8305
D
SG: p=0.5339
T: p<0.0001
T x SG: p=0.0456
% Inactivity
Acclimation Trial Rest
Cued Test
E
SG: p=0.1861
T: p<0.0001
T x SG: p=0.5943
Conditioning Context Cued
Acclimation
F
SG: p=0.2545
T: p=0.3208
T x SG: p=0.3189
Discrimination Index

Figure S7

WT-WD

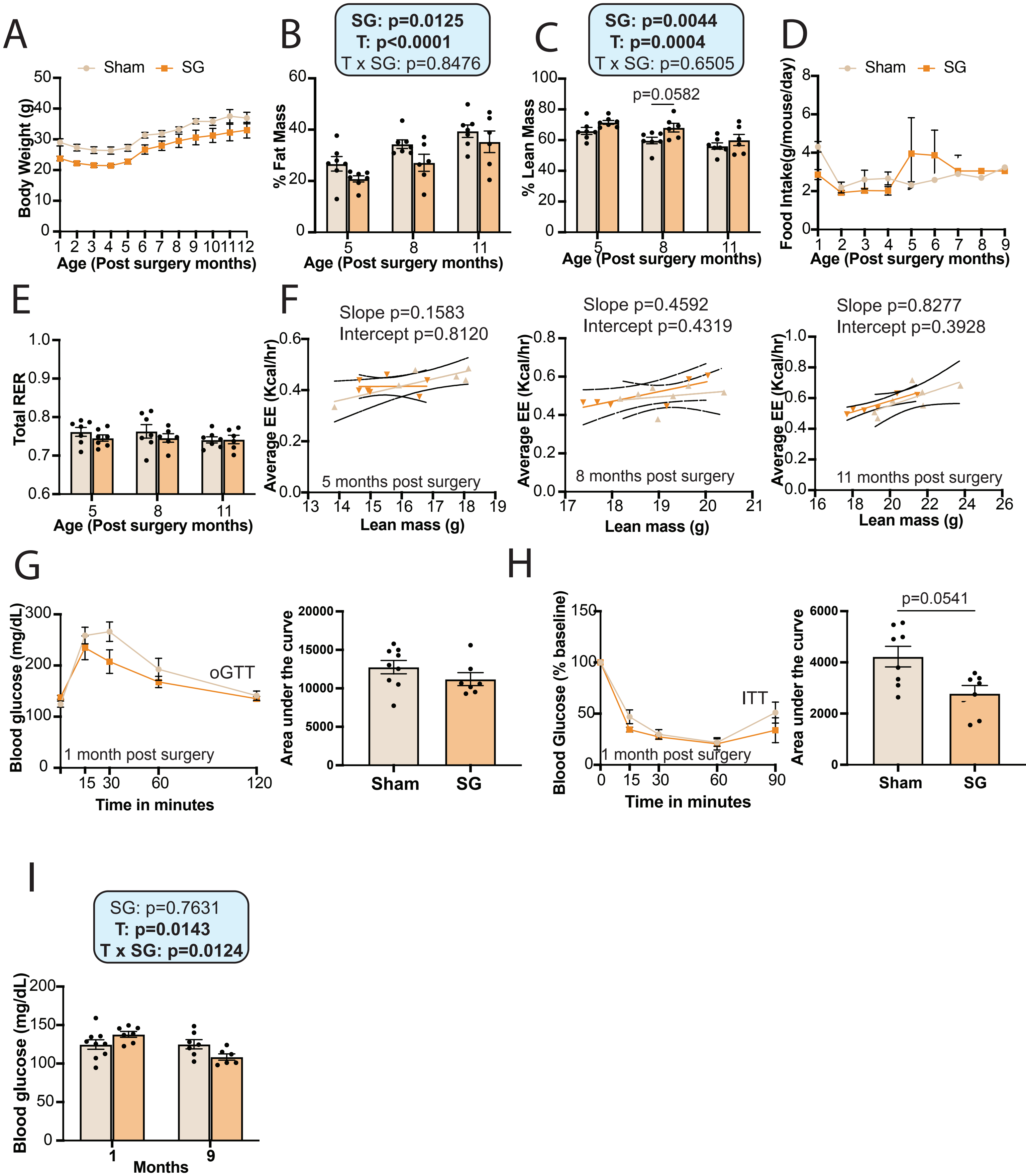

Figure S8

3xTg-WD

A

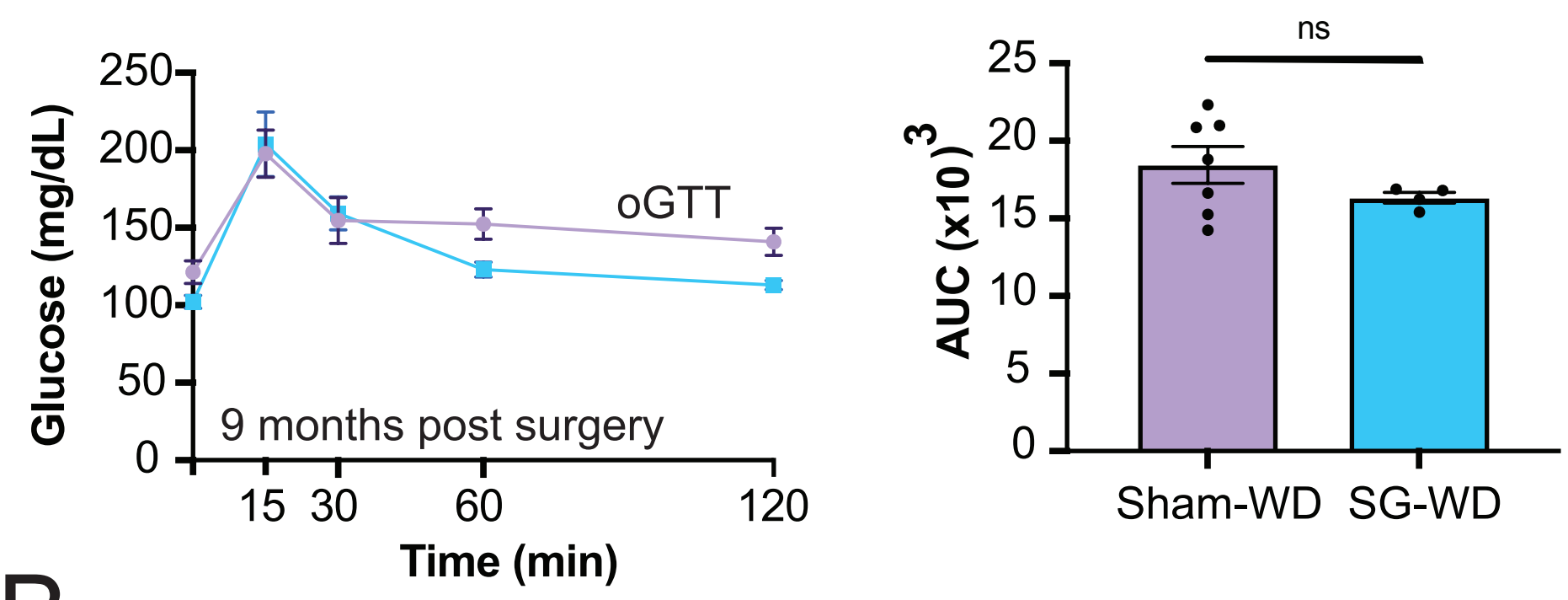

B

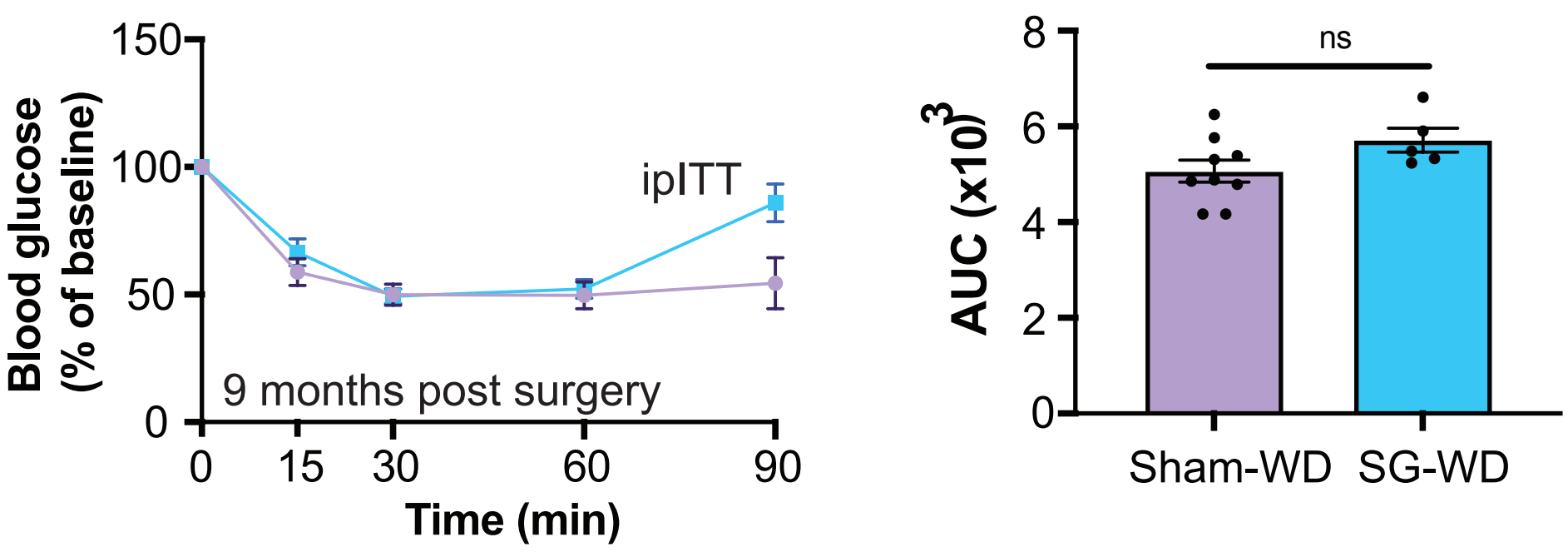

Figure S9

WT-WD

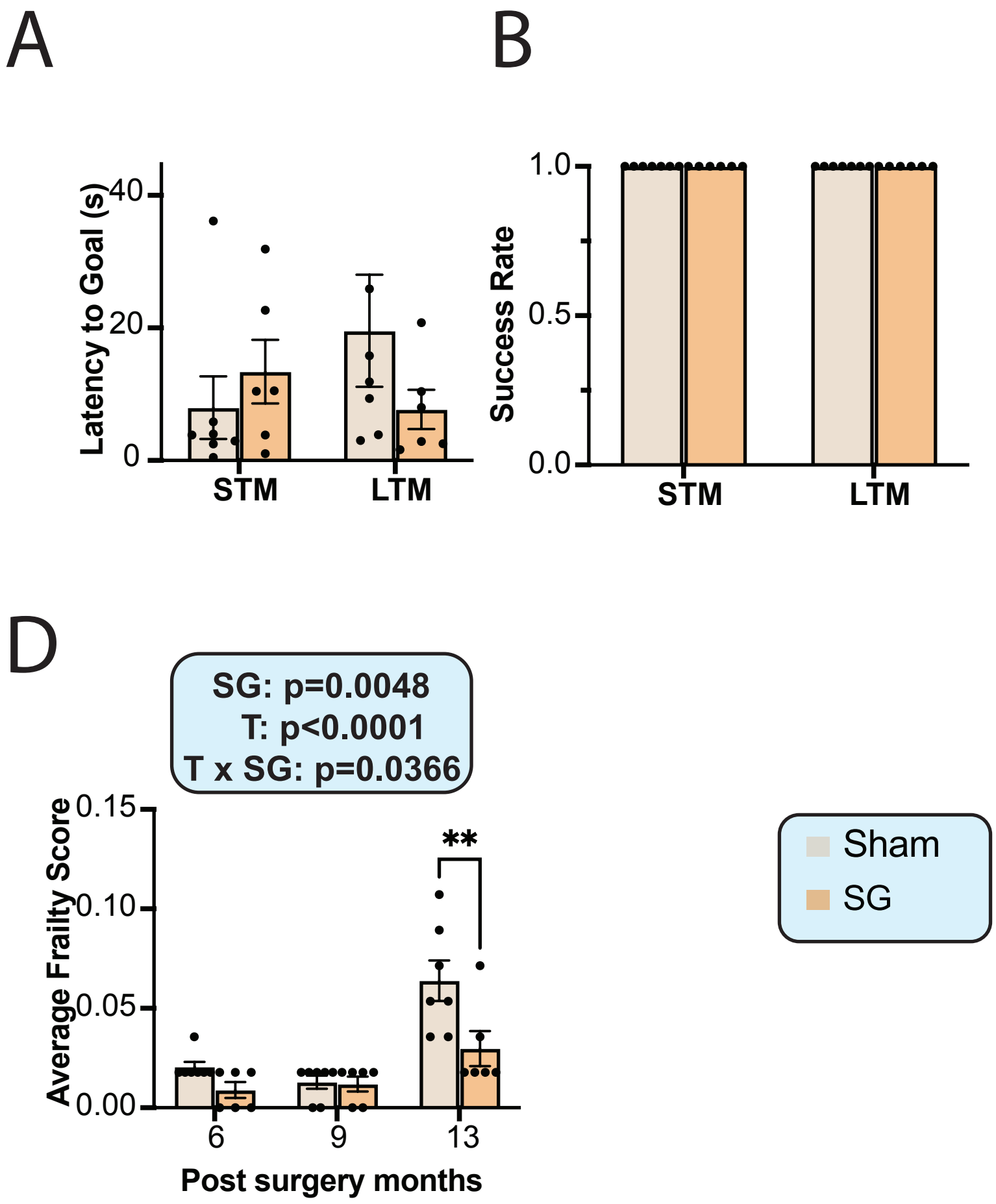

Figure S10

3xTg

A

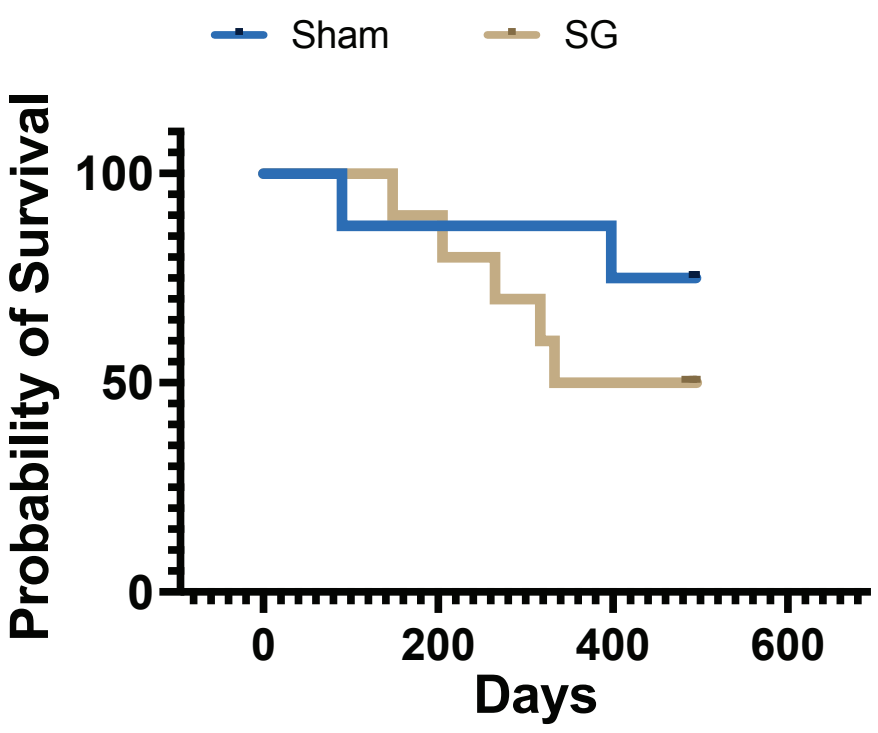

B

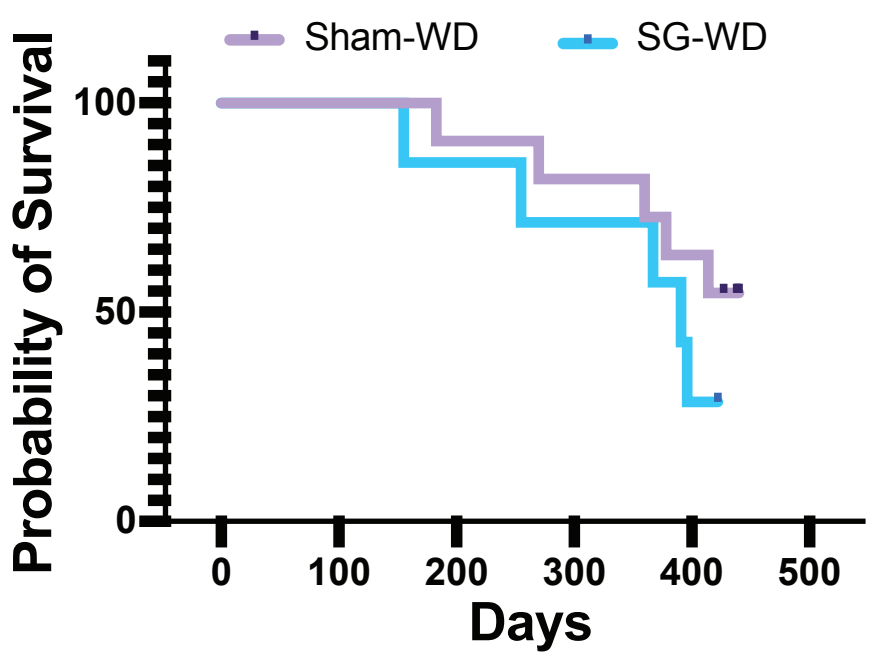

Supplement: Supplementary file 1 [file ACEL-25-e70767-s001.pdf]
